# Relapse and its modifiers in major depressive disorder after antidepressant discontinuation: meta-analysis and meta-regression

**DOI:** 10.1038/s41380-022-01920-0

**Published:** 2022-12-23

**Authors:** Taro Kishi, Kenji Sakuma, Masakazu Hatano, Makoto Okuya, Yuki Matsuda, Masaki Kato, Nakao Iwata

**Affiliations:** 1 Department of Psychiatry, Fujita Health University School of Medicine, Toyoake, Aichi 470-1192 Japan; 2 Department of Clinical Pharmacy, Fujita Health University School of Medicine, Toyoake, Aichi 470–1192 Japan; 3 Department of Psychiatry, Jikei University School of Medicine, Minato-ku, Tokyo 105-8461 Japan; 4 Department of Neuropsychiatry, Kansai Medical University, Hirakata, Osaka 573-1191 Japan

**Keywords:** Depression, Drug discovery

Recent pairwise meta-analysis including only double-blind, randomized placebo-controlled trials (DBRPCTs) with an enrichment design in which individuals with MDD were stabilized on the antidepressant of interest during the open-label study and then randomized to receive the same antidepressant or a placebo was conducted [1]. The meta-analysis reported that the antidepressant maintenance group had a significantly lower relapse rate than the antidepressant discontinuation group (*K* = 40, *n* = 8890) [1]. A subgroup meta-analysis revealed similar relapse rates for maintenance periods of 6 months and 1 year [1]. Therefore, the authors recommended antidepressant maintenance treatment for at least 6 months after remission to prevent relapse [1]. A subsequent DBRPCT found relapse rates for an antidepressant maintenance group and a discontinuation group at 26 weeks of 32 and 56% (24% difference), respectively; and relapse rates at 39 weeks of 40 and 69% (29% difference), respectively [2]. This means there was a 5% difference in relapse rates between the two groups from 26 to 39 weeks. Thus, the differences in relapse rates between individuals with MDD who continued antidepressants that were effective during acute treatment and those who discontinued the antidepressants are still unclear. Trends in the magnitude of the benefit of medication maintenance over time are important in determining how long individuals with MDD should continue taking antidepressants. While the meta-analysis described above was conducted using the relapse rate at the endpoint of each DBRPCT for individuals with MDD including general adults, children and adolescents or older individuals (range of study duration: 14–100 weeks) [1], the current systematic review and pairwise meta-analysis included DBRPCTs with adult participants only and compared relapse rates at matched observation time points (i.e., 3, 6 [primary outcome], 9, 12, 15, and 18 months) between discontinuation and maintenance groups to more accurately determine the temporal relapse trend.

Other recent meta-analyses for acute depression reported a correlation between antidepressant dosage or the type of antidepressant and efficacy [3, 4]. However, it remains unknown which clinical factors are associated with antidepressant efficacy in adults with MDD during the maintenance phase. In the current meta-regression analyses, we attempted to identify the variables in participants, treatment, and/or study design that influence the effect size for the primary outcome. Our meta-regression also aimed to identify modifiers of the antidepressant response and the interplay between these modifiers and the placebo response in the formation of effect sizes.

This systematic review and pairwise meta-analysis was conducted according to the Preferred Reporting Items for Systematic Reviews and Meta-Analyses statement (Table S1) [5], and was registered with the Open Science Framework (https://osf.io/9wnze/). Table S2 presents the definitions of relapse/recurrence used by each included study. Table S3 shows the results of the data synthesis. This pairwise meta-analysis used a random-effects model [6]. We calculated risk ratios (RRs) with 95% confidence intervals (95% CIs). We assessed the heterogeneity of the included studies using the *I*^2^ statistic, with an *I*^2^ of ≥50% indicating heterogeneity [7]. We also conducted a single-group summary meta-analysis to determine the exact relapse rates with 95% CIs in both the maintenance and discontinuation groups. When the pairwise meta-analysis showed significant differences in the relapse rates between the treatment groups, the number needed to treat to benefit (NNTB) was estimated. We performed all statistical analyses using Comprehensive Meta-Analysis software version 3 (Biostat Inc., Englewood, NJ, USA).

Fig. S1 shows the literature search and selection strategy. We identified 35 DBRPCTs with a total of 9442 adults with MDD (68% female with a mean age of 43.5 years; range of mean age among all 35 studies = 37.0–51.0 years). Table S4 summarizes the characteristics of the included DBRPCTs. The mean study duration was 41.9 ± 17.2 weeks. No studies were found to have a high risk of bias in at least one domain of the Risk of Bias 2 tool (Fig. S2).

The RRs for the relapse rates at 3, 6, 9, and 12 months were similar; however, the NNTB slightly decreased over time (Figs. 1 and  S3–8). Although the average relapse rates in both the maintenance group and discontinuation group increased over time, the rate of relapse increased faster in the discontinuation group than in the maintenance group (Fig. 1). The effect of antidepressants on the prevention of relapse in the maintenance group was greater at 15 and 18 months than at 12 months or less; however, the number of studies showing relapse rates for 15 months and 18 months was small, so these results might not be robust. Nevertheless, this suggests that in individuals with MDD whose acute symptoms are improved by antidepressant treatment, maintenance treatment with antidepressants should be continued for 18 months (or at least 12 months) to prevent relapse. As in the previous meta-analysis [1], we found a significantly lower all-cause discontinuation rate (Fig. S9) in the maintenance group than in the discontinuation group, but no significant difference was observed between the groups in discontinuation due to adverse events (Fig. S10).

**Fig. 1:**
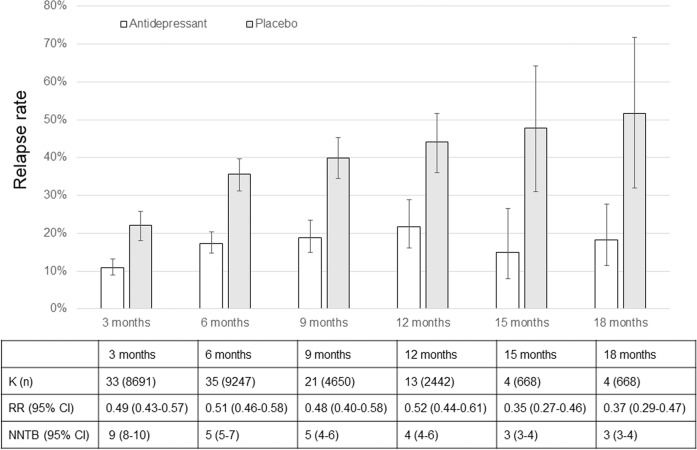
Major depression relapse rates with discontinuation or maintenance of antidepressant treatment. The error bar represents the standard error. 95% CI, 95% confidence interval; K, number of studies; n, number of patients; NNTB number needed to treat to benefit, RR risk ratio.

We found effect size to be correlated with average age (Fig. S11), total number of participants (Fig. S12), dosing schedule (Fig. S13), drug class (Fig. S14), and publication year (Fig. S15 and Table S5). Among these moderators, only average age was associated with the relapse rate in both the maintenance and discontinuation groups, i.e., there were lower relapse rates in both groups for older than younger adults. However, studies including older adults had greater effect sizes than studies including younger adults. Thus, despite their lower relapse rate, older adults might benefit more from continued antidepressant medication than younger adults. These results were confirmed by the multivariate analysis (Table S6). However, it was difficult to identify the reasons for the associations between effect size and the other moderators, since the moderators were not associated with relapse rates in both treatment groups. Our study had several limitations. These are presented in the Supplementary text; e.g., we detected a significant publication bias for the primary outcome (Fig. S16). We also were unable to perform a meta-analysis on those outcomes since data on the relapse rates at time points longer than 18 months of observation was insufficient. Therefore, it remains unclear at this time whether a longer period of antidepressant treatment for those individuals is necessary.

## Supplementary information

Supplementary materials

## Data Availability

The data used for the current study were reported in the articles of the studies included in our meta-analysis.
