# Supplementary material for: Relapse and its modifiers in major depressive disorder after antidepressant discontinuation: meta-analysis and meta-regression
Source: Mol Psychiatry. 2022 Dec 23;28(3):974–6. doi: 10.1038/s41380-022-01920-0 (PMC10005929; doi:10.1038/s41380-022-01920-0)
Supplement: Supplementary file 1 — Supplementary materials [file 41380_2022_1920_MOESM1_ESM.pdf]

Figure S1. Literature search

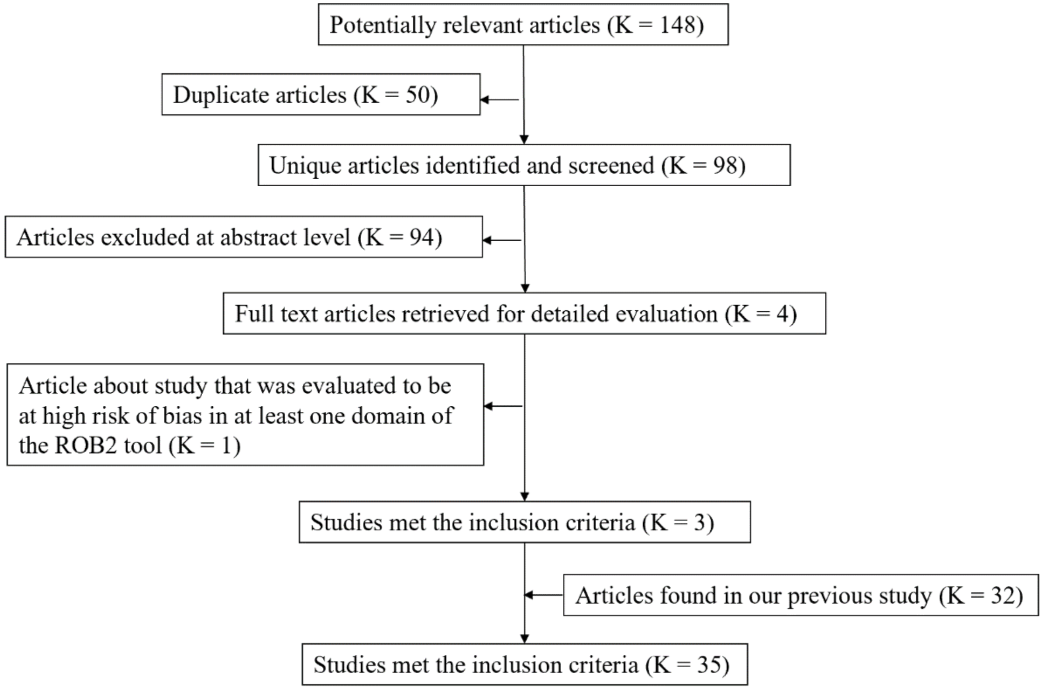

A literature search for previous systematic review and meta-analysis including DBRPCTs with the same inclusion criteria as our study was conducted until June 2018. We have searched PubMed, Cochrane Library, and Embase databases for studies published from January 1, 2018 until May 22, 2022. The search terms for PubMed and Cochrane Library included (major depressi*) AND (random*) AND (double-blind) AND (reccuren* OR relapse) AND (placebo). No language restriction was applied to the literature search. The search terms for Embase included ('major depression'/exp OR 'major depression') AND ('randomized controlled trial'/exp OR 'randomized controlled trial') AND ('placebo'/exp OR placebo) AND ('double blind procedure'/exp OR 'double blind procedure') AND ('relapse' OR 'recurrence'). Relevant studies were identified by searching the Embase, PubMed, and Cochrane Central Register of Controlled Trials databases. The authors evaluated the retrieved studies against the inclusion and exclusion criteria and selected those that were eligible. Furthermore, we manually searched the reference lists of the included studies and reviews for additional relevant published and unpublished research, including conference abstracts. We also searched clinical trial registries, such as ClinicalTrials.gov (http://clinicaltrials.gov/) and the World Health Organization International Clinical Trials Registry Platform (http://www.who.int/ictrp/search/en/) to ensure that the included set of DBRPCTs was comprehensive and to minimize the influence of publication bias. Any discrepancies in the article selection were resolved through consensus discussions among the authors.

Articles included in the previous systematic review (K = 32)

1. Boulenger JP, Loft H, Florea I. A randomized clinical study of Lu AA21004 in the prevention of relapse in patients with major depressive disorder. *J Psychopharmacol* 2012; **26**(11)**:** 1408-1416.
2. Dalery J, Dagens-Lafont V, De Bodinat C. Efficacy of tianeptine vs placebo in the long-term treatment (16.5 months) of unipolar major recurrent depression*.

Hum Psychopharmacol 2001; **16**(S1)**:** S39-S47.

3. Dekker J, Jonghe F, Tuynman H. The use of anti-depressants after recovery from depression. *Eur J Psychiatry* 2000; **14:** 207-212.
4. Dobson KS, Hollon SD, Dimidjian S, Schmaling KB, Kohlenberg RJ, Gallop RJ *et al.* Randomized trial of behavioral activation, cognitive therapy, and antidepressant medication in the prevention of relapse and recurrence in major depression. *J Consult Clin Psychol* 2008; **76**(3)**:** 468-477.
5. Doogan DP, Caillard V. Sertraline in the prevention of depression. *Br J Psychiatry* 1992; **160:** 217-222.
6. Feiger AD, Bielski RJ, Bremner J, Heiser JF, Trivedi M, Wilcox CS *et al.* Double-blind, placebo-substitution study of nefazodone in the prevention of relapse during continuation treatment of outpatients with major depression. *Int Clin Psychopharmacol* 1999; **14**(1)**:** 19-28.
7. Gilaberte I, Montejo AL, de la Gandara J, Perez-Sola V, Bernardo M, Massana J *et al.* Fluoxetine in the prevention of depressive recurrences: a double-blind study. *J Clin Psychopharmacol* 2001; **21**(4)**:** 417-424.
8. Goodwin GM, Boyer P, Emsley R, Rouillon F, de Bodinat C. Is it time to shift to better characterization of patients in trials assessing novel antidepressants? An example of two relapse prevention studies with agomelatine. *Int Clin Psychopharmacol* 2013; **28**(1)**:** 20-28.
9. Goodwin GM, Emsley R, Rembry S, Rouillon F, Agomelatine Study G. Agomelatine prevents relapse in patients with major depressive disorder without evidence of a discontinuation syndrome: a 24-week randomized, double-blind, placebo-controlled trial. *J Clin Psychiatry* 2009; **70**(8)**:** 1128-1137.
10. Hochstrasser B, Isaksen PM, Koponen H, Lauritzen L, Mahnert FA, Rouillon F *et al.* Prophylactic effect of citalopram in unipolar, recurrent depression: placebo-controlled study of maintenance therapy. *Br J Psychiatry* 2001; **178:** 304-310.
11. Keller MB, Kocsis JH, Thase ME, Gelenberg AJ, Rush AJ, Koran L *et al.* Maintenance phase efficacy of sertraline for chronic depression: a randomized controlled trial. *JAMA* 1998; **280**(19)**:** 1665-1672.
12. Kocsis JH, Thase ME, Trivedi MH, Shelton RC, Kornstein SG, Nemeroff CB *et al.* Prevention of recurrent episodes of depression with venlafaxine ER in a 1-year maintenance phase from the PREVENT Study. *J Clin Psychiatry* 2007; **68**(7)**:** 1014-1023.
13. McGrath PJ, Stewart JW, Quitkin FM, Chen Y, Alpert JE, Nierenberg AA *et al.* Predictors of relapse in a prospective study of fluoxetine treatment of major depression. *Am J Psychiatry* 2006; **163**(9)**:** 1542-1548.
14. Montgomery SA, Dunbar G. Paroxetine is better than placebo in relapse prevention and the prophylaxis of recurrent depression. *Int Clin Psychopharmacol* 1993; **8**(3)**:** 189-195.
15. Montgomery SA, Entsuah R, Hackett D, Kunz NR, Rudolph RL, Venlafaxine 335 Study G. Venlafaxine versus placebo in the preventive treatment of recurrent major depression. *J Clin Psychiatry* 2004; **65**(3)**:** 328-336.

16. Montgomery SA, Rasmussen JG, Tanghoj P. A 24-week study of 20 mg citalopram, 40 mg citalopram, and placebo in the prevention of relapse of major depression. *Int Clin Psychopharmacol* 1993; **8**(3)**:** 181-188.

17. Perahia DG, Gilaberte I, Wang F, Wiltse CG, Huckins SA, Clemens JW *et al.* Duloxetine in the prevention of relapse of major depressive disorder: double-blind placebo-controlled study. *Br J Psychiatry* 2006; **188:** 346-353.

18. Perahia DG, Maina G, Thase ME, Spann ME, Wang F, Walker DJ *et al.* Duloxetine in the prevention of depressive recurrences: a randomized, double-blind, placebo-controlled trial. *J Clin Psychiatry* 2009; **70**(5)**:** 706-716.

19. Rapaport MH, Bose A, Zheng H. Escitalopram continuation treatment prevents relapse of depressive episodes. *J Clin Psychiatry* 2004; **65**(1)**:** 44-49.

20. Rickels K, Montgomery SA, Tourian KA, Guelfi JD, Pitrosky B, Padmanabhan SK *et al.* Desvenlafaxine for the prevention of relapse in major depressive disorder: results of a randomized trial. *J Clin Psychopharmacol* 2010; **30**(1)**:** 18-24.

21. Robert P, Montgomery SA. Citalopram in doses of 20-60 mg is effective in depression relapse prevention: a placebo-controlled 6 month study. *Int Clin Psychopharmacol* 1995; **10 Suppl 1:** 29-35.

22. Rosenthal JZ, Boyer P, Vialet C, Hwang E, Tourian KA. Efficacy and safety of desvenlafaxine 50 mg/d for prevention of relapse in major depressive disorder:a randomized controlled trial. *J Clin Psychiatry* 2013; **74**(2)**:** 158-166.

23. Rouillon F, Warner B, Pezous N, Bisserbe JC. Milnacipran efficacy in the prevention of recurrent depression: a 12-month placebo-controlled study. Milnacipran recurrence prevention study group. *Int Clin Psychopharmacol* 2000; **15**(3)**:** 133-140.

24. Schmidt ME, Fava M, Robinson JM, Judge R. The efficacy and safety of a new enteric-coated formulation of fluoxetine given once weekly during the continuation treatment of major depressive disorder. *J Clin Psychiatry* 2000; **61**(11)**:** 851-857.

25. Segal ZV, Bieling P, Young T, MacQueen G, Cooke R, Martin L *et al.* Antidepressant monotherapy vs sequential pharmacotherapy and mindfulness-based cognitive therapy, or placebo, for relapse prophylaxis in recurrent depression. *Arch Gen Psychiatry* 2010; **67**(12)**:** 1256-1264.

26. Shiovitz T, Greenberg WM, Chen C, Forero G, Gommoll CP. A Randomized, Double-blind, Placebo-controlled Trial of the Efficacy and Safety of Levomilnacipran ER 40-120mg/day for Prevention of Relapse in Patients with Major Depressive Disorder. *Innov Clin Neurosci* 2014; **11**(1-2)**:** 10-22.

27. Simon JS, Aguiar LM, Kunz NR, Lei D. Extended-release venlafaxine in relapse prevention for patients with major depressive disorder. *J Psychiatr Res* 2004; **38**(3)**:** 249-257.

28. Stein MK, Rickels K, Weise CC. Maintenance therapy with amitriptyline: a controlled trial. *Am J Psychiatry* 1980; **137**(3)**:** 370-371.

29. Terra JL, Montgomery SA. Fluvoxamine prevents recurrence of depression: results of a long-term, double-blind, placebo-controlled study. *Int Clin*

Psychopharmacol 1998; **13**(2)**:** 55-62.

30. Thase ME, Nierenberg AA, Keller MB, Panagides J, Relapse Prevention Study G. Efficacy of mirtazapine for prevention of depressive relapse: a placebo-controlled double-blind trial of recently remitted high-risk patients. *J Clin Psychiatry* 2001; **62**(10)**:** 782-788.
31. Versiani M, Mehilane L, Gaszner P, Arnaud-Castiglioni R. Reboxetine, a unique selective NRI, prevents relapse and recurrence in long-term treatment of major depressive disorder. *J Clin Psychiatry* 1999; **60**(6)**:** 400-406.
32. Weihs KL, Houser TL, Batey SR, Ascher JA, Bolden-Watson C, Donahue RM *et al.* Continuation phase treatment with bupropion SR effectively decreases the risk for relapse of depression. *Biol Psychiatry* 2002; **51**(9)**:** 753-761.

Articles included in the previous systematic review but not in our study (K = 2)

1. Reimherr FW, Amsterdam JD, Quitkin FM, Rosenbaum JF, Fava M, Zajecka J *et al.* Optimal length of continuation therapy in depression: a prospective assessment during long-term fluoxetine treatment. *Am J Psychiatry* 1998; **155**(9)**:** 1247-1253.

Reasons for exclusion: the study included individuals with MDD as well as individuals with BD2.

2. Stewart JW, Tricamo E, McGrath PJ, Quitkin FM. Prophylactic efficacy of phenelzine and imipramine in chronic atypical depression: likelihood of recurrence on discontinuation after 6 months' remission. *Am J Psychiatry* 1997; **154**(1)**:** 31-36.

Reasons for exclusion: the study included individuals with MDD as well as individuals with BD2.

Article which we have excluded from the meta-analysis (K = 1)

1. Lewis G, Marston L, Duffy L, Freemantle N, Gilbody S, Hunter R et al. Maintenance or Discontinuation of Antidepressants in Primary Care. N Engl J Med 2021; 385(14): 1257-1267.

Reasons for exclusion: the study was evaluated to be at high risk of bias in at least one domain of the ROB2 tool.

Articles which we have found in the current literature search (K = 3)

1. Durgam S, Chen C, Migliore R, Prakash C, Thase ME. Relapse prevention with levomilnacipran ER in adults with major depressive disorder: A multicenter, randomized, double-blind, placebo-controlled study. *Depress Anxiety* 2019; **36**(3)**:** 225-234.
2. Durgam S, Gommoll C, Migliore R, Chen C, Chang CT, Aguirre M *et al.* Relapse prevention in adults with major depressive disorder treated with vilazodone: a

randomized, double-blind, placebo-controlled trial. *Int Clin Psychopharmacol* 2018; **33**(6)**:** 304-311.

3. Thase ME, Jacobsen PL, Hanson E, Xu R, Tolkoff M, Murthy NV. Vortioxetine 5, 10, and 20 mg significantly reduces the risk of relapse compared with placebo in patients with remitted major depressive disorder: The RESET study. *J Affect Disord* 2022; **303:** 123-130.

Figure S2. Risk of bias summary

	Randomization process	Deviation from intended intervention	Missing outcome data	Measurement of the outcome	Selection of the reported result	Overall risk of bias
Stein 1980	Some concerns	Some concerns	Low	Some concerns	Low	Some concerns
Doogan 1992	Some concerns	Low	Low	Some concerns	Low	Some concerns
Montgomery 1993a PAR	Some concerns	Some concerns	Low	Some concerns	Low	Some concerns
Montgomery 1993b CIT	Some concerns	Some concerns	Low	Some concerns	Low	Some concerns
Robert 1995	Some concerns	Some concerns	Low	Some concerns	Low	Some concerns
Keller 1998	Some concerns	Low	Low	Low	Low	Some concerns
Terra 1998	Some concerns	Some concerns	Low	Some concerns	Low	Some concerns
Feiger 1999	Some concerns	Low	Low	Some concerns	Low	Some concerns
Versiani 1999	Some concerns	Some concerns	Low	Some concerns	Low	Some concerns
Dekker 2000	Some concerns*	Low	Low	Low	Low	Some concerns
Rouillon 2000	Some concerns	Some concerns	Low	Some concerns	Low	Some concerns
Schmidt 2000	Some concerns	Some concerns	Low	Some concerns	Low	Some concerns
Dalery 2001	Some concerns	Low	Low	Some concerns	Low	Some concerns
Gilaberte 2001	Some concerns	Some concerns	Low	Some concerns	Low	Some concerns
Hochstrasser 2001	Some concerns	Some concerns	Low	Some concerns	Low	Some concerns
Thase 2001	Some concerns	Low	Low	Some concerns	Low	Some concerns
Weihs 2002	Some concerns	Low	Low	Some concerns	Low	Some concerns
Montgomery 2004	Some concerns	Some concerns	Low	Some concerns	Low	Some concerns
Rapaport 2004	Some concerns	Some concerns	Low	Some concerns	Low	Some concerns
Simon 2004	Some concerns	Some concerns	Low	Some concerns	Low	Some concerns
Perahia 2006	Some concerns	Some concerns	Low	Some concerns	Low	Some concerns
McGrath 2006	Some concerns*	Low	Low	Low	Low	Some concerns
Kocsis 2007	Some concerns	Low	Low	Low	Low	Some concerns
Dobson 2008	Some concerns*	Some concerns	Low	Some concerns	Low	Some concerns
Goodwin 2009	Some concerns*	Low	Low	Low	Low	Some concerns
Perahia 2009	Some concerns	Some concerns	Low	Some concerns	Low	Some concerns
Rickels 2010	Some concerns	Some concerns	Low	Some concerns	Low	Some concerns
Segal 2010	Low	Low	Low	Low	Low	Low risk
Boulenger 2012	Low	Low	Low	Low	Low	Low risk
Goodwin 2013	Some concerns*	Low	Low	Low	Low	Some concerns
Rosenthal 2013	Some concerns*	Some concerns	Low	Some concerns	Low	Some concerns
Shiovitz 2014	Low	Low	Low	Low	Low	Low risk

Durgam 2018	Low	Low	Low	Low	Low	Low risk
Durgam 2019	Low	Low	Low	Low	Low	Low risk
Thase 2022	Low	Low	Low	Low	Low	Low risk

*We did not find sufficient information to assess the risk of bias with respect to “allocation concealment.”

“Some concerns” in each domain: we did not have sufficient information to assess the risk of bias with respect to the domains.

Figure S3. Forest plot for relapse rate at 3 months

Study name	Statistics for each study				
	Risk ratio	Lower limit	Upper limit	Z-Value	p-Value
Doogan 1992	0.127	0.054	0.298	-4.753	0.000
Montgomery 1993 PAR	0.164	0.038	0.706	-2.428	0.015
Montgomery 1993 CIT	0.267	0.117	0.605	-3.160	0.002
Robert 1995	0.664	0.321	1.373	-1.105	0.269
Keller 1998	0.109	0.014	0.832	-2.137	0.033
Terra 1998	0.172	0.061	0.487	-3.320	0.001
Feiger 1999	0.290	0.063	1.345	-1.581	0.114
Versiani 1999	0.709	0.382	1.314	-1.092	0.275
Dekker 2000	0.571	0.211	1.551	-1.099	0.272
Rouillon 2000	0.740	0.293	1.873	-0.635	0.526
Dalery 2001	0.056	0.007	0.418	-2.806	0.005
Gilaberte 2001	0.500	0.253	0.990	-1.989	0.047
Hochstrasser 2001	0.067	0.004	1.156	-1.861	0.063
Thase 2001	0.436	0.240	0.790	-2.738	0.006
Weihs 2002	0.681	0.493	0.940	-2.338	0.019
Montgomery 2004	0.405	0.243	0.677	-3.449	0.001
Rapaport 2004	0.637	0.401	1.013	-1.905	0.057
Simon 2004	0.448	0.311	0.645	-4.313	0.000
Perahia 2006	0.586	0.372	0.922	-2.313	0.021
McGrath 2006	0.360	0.222	0.583	-4.161	0.000
Kocsis 2007	0.531	0.311	0.907	-2.316	0.021
Dobson 2008	0.583	0.260	1.311	-1.305	0.192
Goodwin 2009	0.561	0.363	0.867	-2.599	0.009
Perahia 2009	0.292	0.144	0.592	-3.409	0.001
Rickels 2010	0.518	0.365	0.735	-3.687	0.000
Segal 2010	0.412	0.169	1.007	-1.944	0.052
Boulenger 2012	0.471	0.043	5.148	-0.618	0.537
Goodwin 2013	0.797	0.505	1.257	-0.976	0.329
Rosenthal 2013	0.518	0.326	0.823	-2.787	0.005
Shiovitz 2014	0.787	0.409	1.513	-0.720	0.472

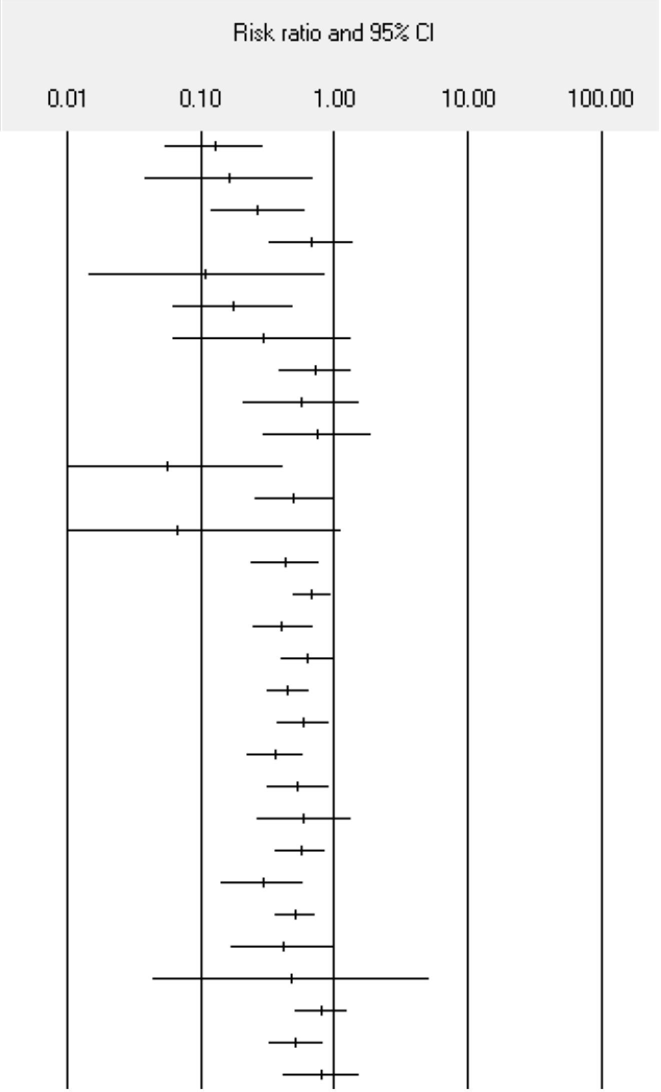

Durgam 2018	1.024	0.539	1.947	0.073	0.942
Durgam 2019	0.526	0.309	0.894	-2.372	0.018
Thase 2022	0.426	0.289	0.628	-4.314	0.000
	0.493	0.428	0.567	-9.895	0.000

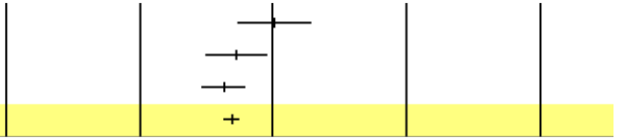

$I^2 = 38.77\%$

Figure S4. Forest plot for relapse rate at 6 months

Study name	Statistics for each study				
	Risk ratio	Lower limit	Upper limit	Z-Value	p-Value
Stein 1980	0.398	0.209	0.758	-2.805	0.005
Doogan 1992	0.152	0.082	0.282	-5.965	0.000
Montgomery 1993 PAR	0.235	0.094	0.586	-3.105	0.002
Montgomery 1993 CIT	0.314	0.155	0.635	-3.223	0.001
Robert 1995	0.603	0.364	0.997	-1.971	0.049
Keller 1998	0.218	0.078	0.610	-2.903	0.004
Terra 1998	0.300	0.141	0.639	-3.123	0.002
Feiger 1999	0.169	0.039	0.727	-2.389	0.017
Versiani 1999	0.523	0.358	0.765	-3.341	0.001
Dekker 2000	0.500	0.191	1.310	-1.410	0.159
Rouillon 2000	0.721	0.396	1.313	-1.070	0.285
Schmidt 2000	0.628	0.498	0.792	-3.938	0.000
Dalery 2001	0.267	0.108	0.656	-2.879	0.004
Gilaberte 2001	0.379	0.206	0.698	-3.115	0.002
Hochstrasser 2001	0.310	0.194	0.497	-4.873	0.000
Thase 2001	0.409	0.241	0.697	-3.293	0.001
Weihs 2002	0.725	0.562	0.934	-2.482	0.013
Montgomery 2004	0.418	0.275	0.635	-4.088	0.000
Rapaport 2004	0.604	0.412	0.886	-2.578	0.010
Simon 2004	0.610	0.460	0.808	-3.449	0.001
Perahia 2006	0.503	0.335	0.753	-3.333	0.001
McGrath 2006	0.667	0.507	0.876	-2.907	0.004
Kocsis 2007	0.667	0.433	1.027	-1.839	0.066
Dobson 2008	0.625	0.336	1.162	-1.486	0.137
Goodwin 2009	0.487	0.349	0.679	-4.244	0.000
Perahia 2009	0.384	0.221	0.666	-3.404	0.001
Rickels 2010	0.583	0.444	0.766	-3.874	0.000
Segal 2010	0.335	0.141	0.793	-2.488	0.013
Boulenger 2012	0.555	0.345	0.894	-2.423	0.015
Goodwin 2013	1.035	0.721	1.486	0.187	0.852

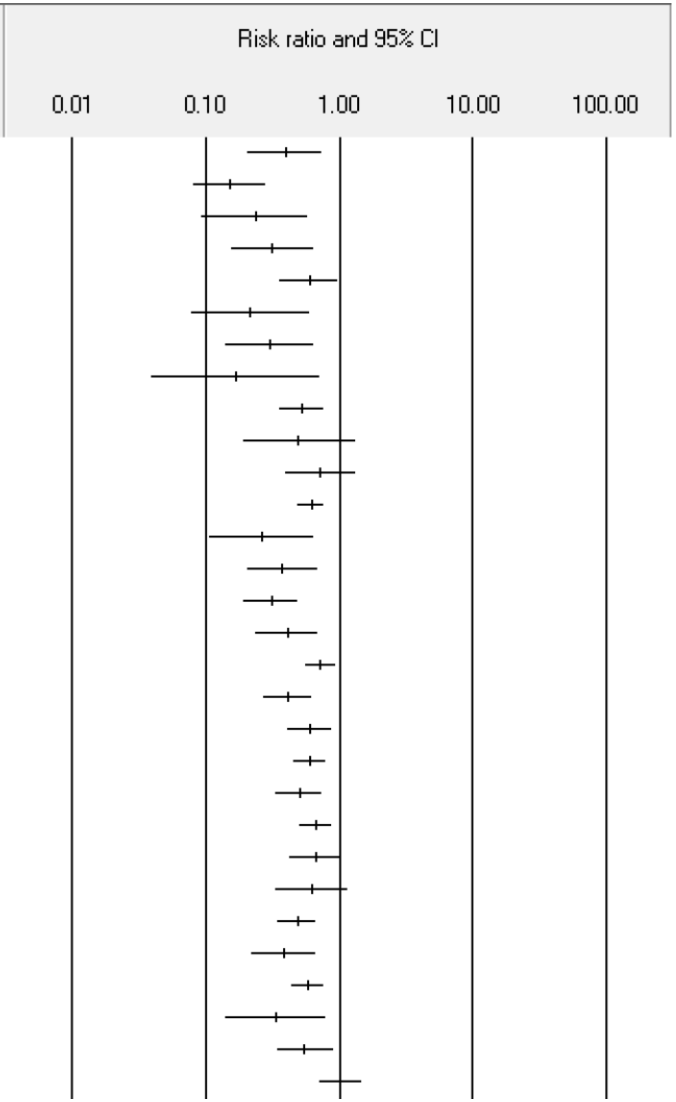

Rosenthal 2013	0.465	0.329	0.656	-4.350	0.000
Shiovitz 2014	0.682	0.430	1.081	-1.629	0.103
Durgam 2018	0.983	0.627	1.543	-0.073	0.942
Durgam 2019	0.564	0.358	0.888	-2.471	0.013
Thase 2022	0.494	0.372	0.657	-4.856	0.000
	0.514	0.457	0.577	-11.189	0.000

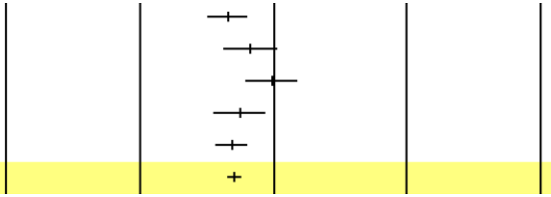

$I^2 = 56.65\%$

Figure S5. Forest plot for relapse rate at 9 months

Study name	Statistics for each study				
	Risk ratio	Lower limit	Upper limit	Z-Value	p-Value
Doogan 1992	0.240	0.149	0.388	-5.849	0.000
Montgomery 1993 PAR	0.394	0.206	0.756	-2.803	0.005
Keller 1998	0.260	0.103	0.655	-2.856	0.004
Terra 1998	0.316	0.168	0.596	-3.563	0.000
Feiger 1999	0.169	0.039	0.727	-2.389	0.017
Versiani 1999	0.402	0.288	0.561	-5.368	0.000
Rouillon 2000	0.718	0.428	1.204	-1.257	0.209
Dalery 2001	0.254	0.119	0.543	-3.538	0.000
Gilaberte 2001	0.382	0.221	0.660	-3.448	0.001
Hochstrasser 2001	0.361	0.236	0.551	-4.719	0.000
Thase 2001	0.471	0.292	0.760	-3.088	0.002
Weihs 2002	0.739	0.578	0.944	-2.423	0.015
Montgomery 2004	0.415	0.277	0.622	-4.261	0.000
Rapaport 2004	0.680	0.481	0.962	-2.181	0.029
McGrath 2006	0.684	0.529	0.884	-2.900	0.004
Kocsis 2007	0.596	0.411	0.865	-2.727	0.006
Dobson 2008	0.688	0.381	1.241	-1.243	0.214
Perahia 2009	0.407	0.247	0.671	-3.527	0.000
Segal 2010	0.417	0.206	0.843	-2.434	0.015
Boulenger 2012	0.505	0.337	0.759	-3.295	0.001
Goodwin 2013	1.005	0.729	1.385	0.029	0.977
	0.482	0.404	0.575	-8.125	0.000

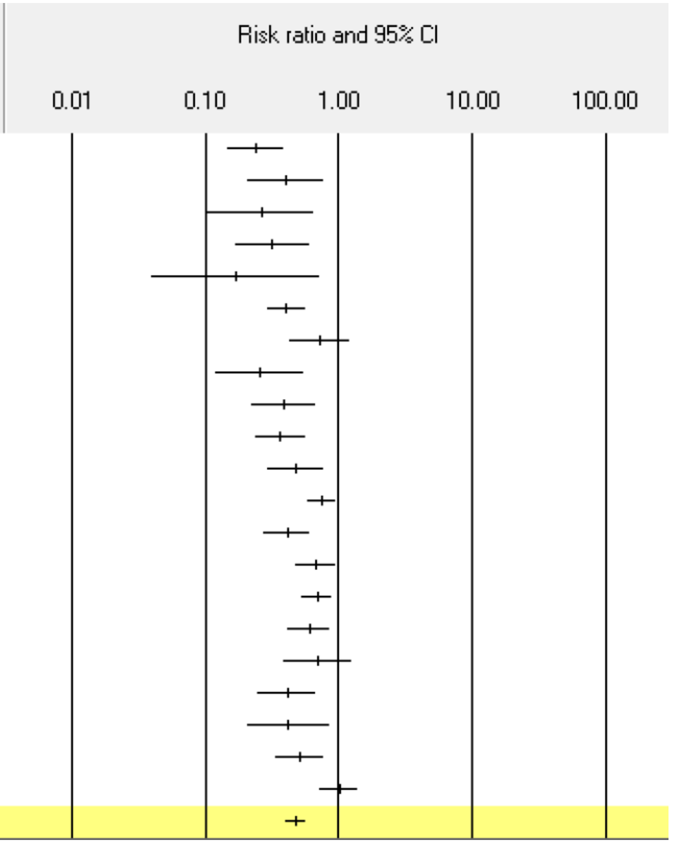

$I^2 = 68.20\%$

Figure S6. Forest plot for relapse rate at 12 months

Study name	Statistics for each study				
	Risk ratio	Lower limit	Upper limit	Z-Value	p-Value
Montgomery 1993 PAR	0.427	0.245	0.745	-2.997	0.003
Keller 1998	0.260	0.103	0.655	-2.856	0.004
Terra 1998	0.431	0.253	0.735	-3.094	0.002
Rouillon 2000	0.648	0.392	1.073	-1.685	0.092
Dalery 2001	0.306	0.159	0.585	-3.574	0.000
Gilaberte 2001	0.471	0.287	0.771	-2.995	0.003
Hochstrasser 2001	0.400	0.272	0.588	-4.658	0.000
Montgomery 2004	0.532	0.381	0.743	-3.700	0.000
McGrath 2006	0.705	0.567	0.875	-3.167	0.002
Kocsis 2007	0.582	0.406	0.835	-2.940	0.003
Dobson 2008	0.813	0.472	1.399	-0.749	0.454
Perahia 2009	0.486	0.308	0.768	-3.094	0.002
Segal 2010	0.459	0.255	0.826	-2.599	0.009
	0.518	0.444	0.605	-8.308	0.000

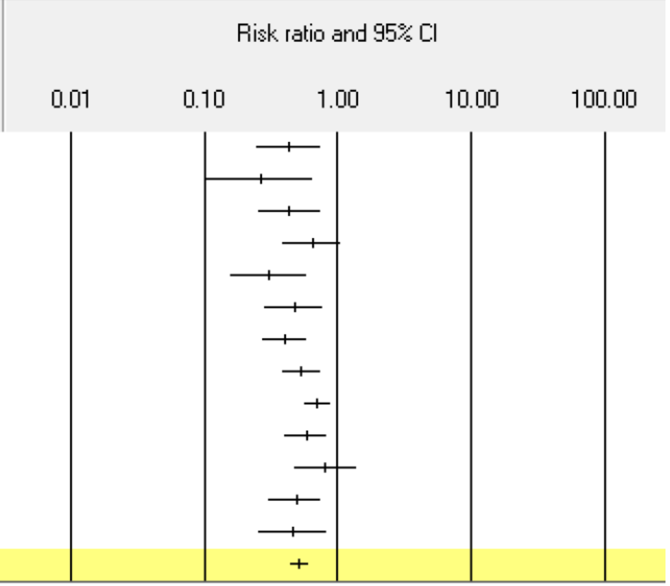

$I^2 = 34.90\%$

Figure S7. Forest plot for relapse rate at 15 months

Study name	Statistics for each study				
	Risk ratio	Lower limit	Upper limit	Z-Value	p-Value
Keller 1998	0.218	0.088	0.542	-3.283	0.001
Dalery 2001	0.272	0.144	0.513	-4.014	0.000
Hochstrasser 2001	0.388	0.265	0.569	-4.841	0.000
Segal 2010	0.402	0.228	0.708	-3.151	0.002
	0.348	0.266	0.457	-7.622	0.000

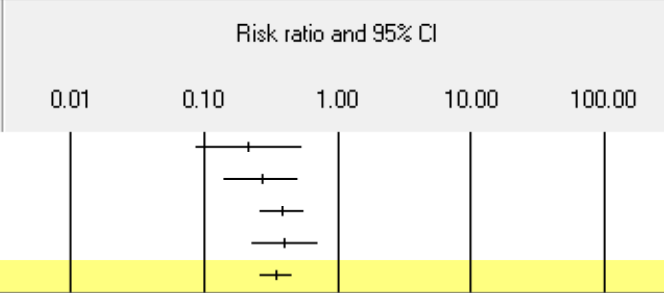

$I^2 = 0.00\%$

Figure S8. Forest plot for relapse rate at 18 months

Study name	Statistics for each study				
	Risk ratio	Lower limit	Upper limit	Z-Value	p-Value
Keller 1998	0.349	0.168	0.727	-2.812	0.005
Dalery 2001	0.370	0.212	0.647	-3.486	0.000
Hochstrasser 2001	0.366	0.260	0.515	-5.769	0.000
Segal 2010	0.402	0.228	0.708	-3.151	0.002
	0.371	0.291	0.474	-7.948	0.000

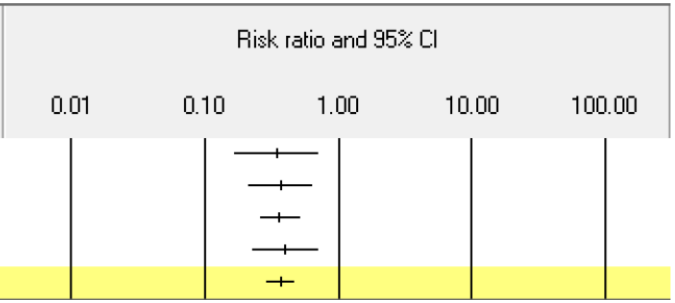

I^2 = 0.00%

Figure S9. Forest plot for all-cause discontinuation

Study name	Risk ratio	Lower limit	Upper limit	Z-Value	p-Value
Doogan 1992	0.619	0.499	0.767	-4.382	0.000
Montgomery 1993 PAR	0.523	0.323	0.847	-2.633	0.008
Keller 1998	0.764	0.598	0.975	-2.160	0.031
Feiger 1999	0.857	0.585	1.254	-0.796	0.426
Versiani 1999	0.844	0.668	1.068	-1.413	0.158
Dekker 2000	0.857	0.376	1.951	-0.367	0.713
Rouillon 2000	0.725	0.526	0.999	-1.965	0.049
Schmidt 2000	0.829	0.720	0.954	-2.623	0.009
Dalery 2001	0.667	0.508	0.874	-2.930	0.003
Gilaberte 2001	0.512	0.340	0.771	-3.210	0.001
Weihs 2002	0.783	0.649	0.945	-2.545	0.011
Montgomery 2004	0.661	0.536	0.816	-3.848	0.000
Rapaport 2004	0.738	0.600	0.907	-2.891	0.004
Simon 2004	0.695	0.582	0.831	-3.986	0.000
Perahia 2006	0.931	0.620	1.398	-0.344	0.731
McGrath 2006	1.500	1.046	2.150	2.207	0.027
Kocsis 2007	0.689	0.564	0.841	-3.661	0.000
Dobson 2008	0.516	0.307	0.866	-2.502	0.012
Goodwin 2009	0.635	0.481	0.840	-3.189	0.001
Perahia 2009	0.705	0.532	0.934	-2.438	0.015
Rickels 2010	0.559	0.435	0.719	-4.529	0.000
Segal 2010	0.893	0.665	1.199	-0.754	0.451
Boulenger 2012	0.848	0.675	1.064	-1.426	0.154
Goodwin 2013	0.980	0.716	1.343	-0.123	0.902
Rosenthal 2013	0.629	0.481	0.823	-3.377	0.001
Shiovitz 2014	1.346	0.851	2.130	1.271	0.204
Durgam 2018	1.207	0.931	1.566	1.421	0.155
Durgam 2019	1.044	0.627	1.739	0.165	0.869
Thase 2022	0.708	0.585	0.858	-3.533	0.000
	0.764	0.706	0.826	-6.729	0.000

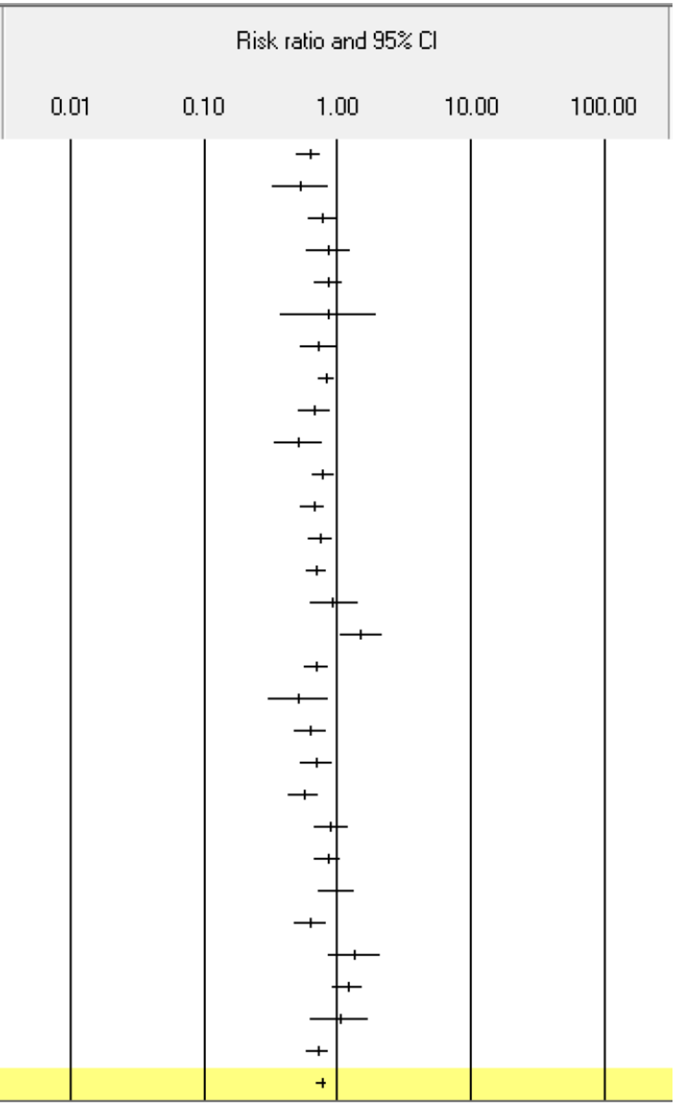

$I^2 = 58.70\%$

Figure S10. Forest plot for discontinuation due to adverse events

Study name	Statistics for each study				
	Risk ratio	Lower limit	Upper limit	Z-Value	p-Value
Doogan 1992	2.081	0.440	9.841	0.925	0.355
Montgomery 1993 PAR	1.642	0.409	6.600	0.699	0.485
Keller 1998	8.727	1.117	68.182	2.066	0.039
Terra 1998	4.279	0.208	88.038	0.942	0.346
Feiger 1999	0.338	0.036	3.170	-0.949	0.343
Versiani 1999	2.917	0.599	14.212	1.325	0.185
Dekker 2000	5.000	0.260	96.127	1.067	0.286
Schmidt 2000	1.931	0.438	8.510	0.870	0.384
Dalery 2001	4.688	0.246	89.445	1.027	0.304
Gilaberte 2001	0.200	0.010	4.092	-1.045	0.296
Thase 2001	4.737	1.057	21.222	2.033	0.042
Weihs 2002	4.057	0.872	18.882	1.785	0.074
Montgomery 2004	0.732	0.269	1.992	-0.611	0.541
Rapaport 2004	0.514	0.186	1.421	-1.283	0.200
Simon 2004	0.670	0.321	1.399	-1.066	0.287
Perahia 2006	1.044	0.309	3.526	0.070	0.945
Kocsis 2007	0.372	0.121	1.139	-1.733	0.083
Goodwin 2009	4.218	0.476	37.350	1.294	0.196
Perahia 2009	1.945	0.496	7.628	0.954	0.340
Rickels 2010	0.620	0.373	1.030	-1.846	0.065
Boulenger 2012	3.014	1.126	8.069	2.195	0.028
Goodwin 2013	0.321	0.034	3.056	-0.989	0.323
Rosenthal 2013	0.290	0.061	1.383	-1.553	0.120
Shiovitz 2014	1.282	0.347	4.742	0.373	0.709
Durgam 2018	1.290	0.253	6.589	0.306	0.759
Durgam 2019	2.409	0.474	12.238	1.060	0.289
Thase 2022	1.232	0.412	3.685	0.373	0.709
	1.229	0.874	1.727	1.185	0.236

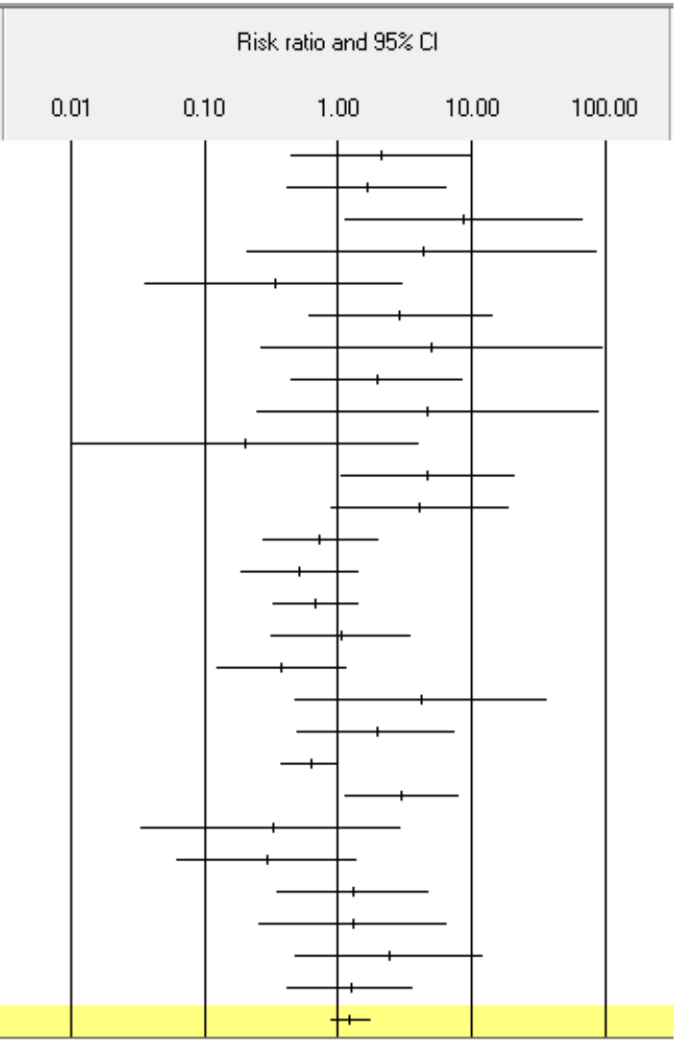

$I^2 = 38.47\%$

Figure S11. Bubble plot: average age

Figure S11-1. For effect size

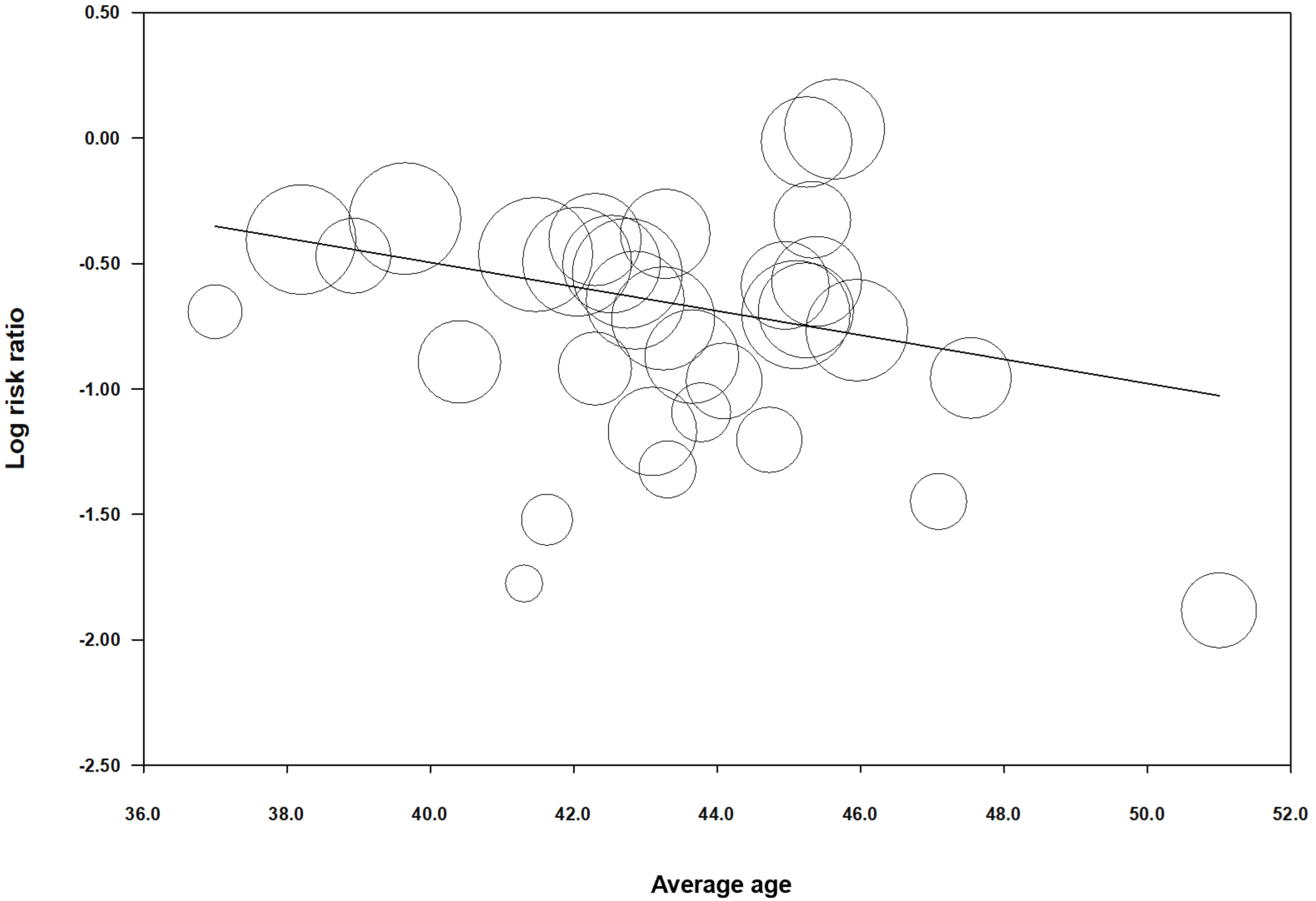

Figure S11-2. For event rate in each treatment group

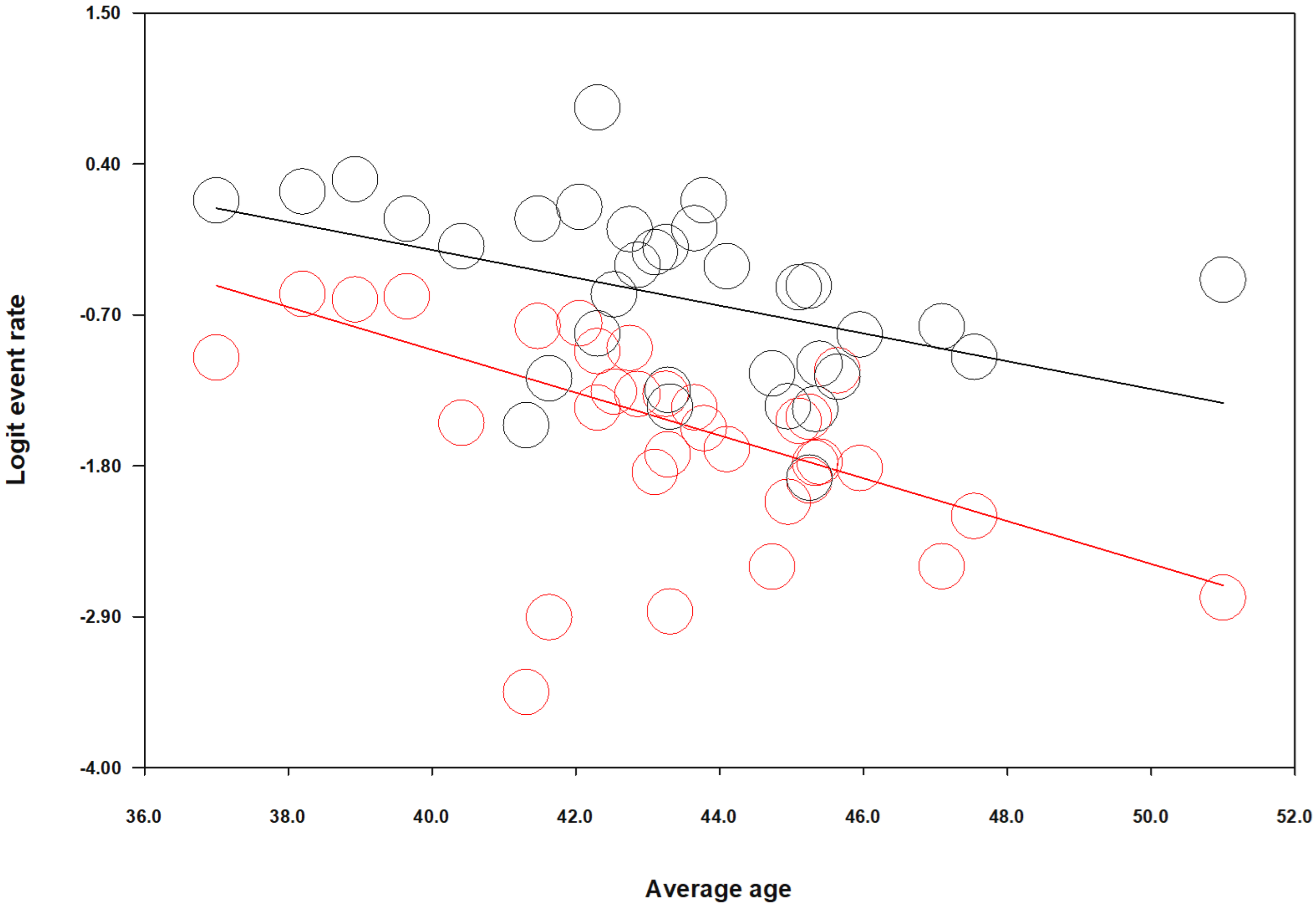

Red line, maintenance group; black line, discontinuation group

Figure S12. Bubble plot: total number of participants

Figure S12-1. For effect size

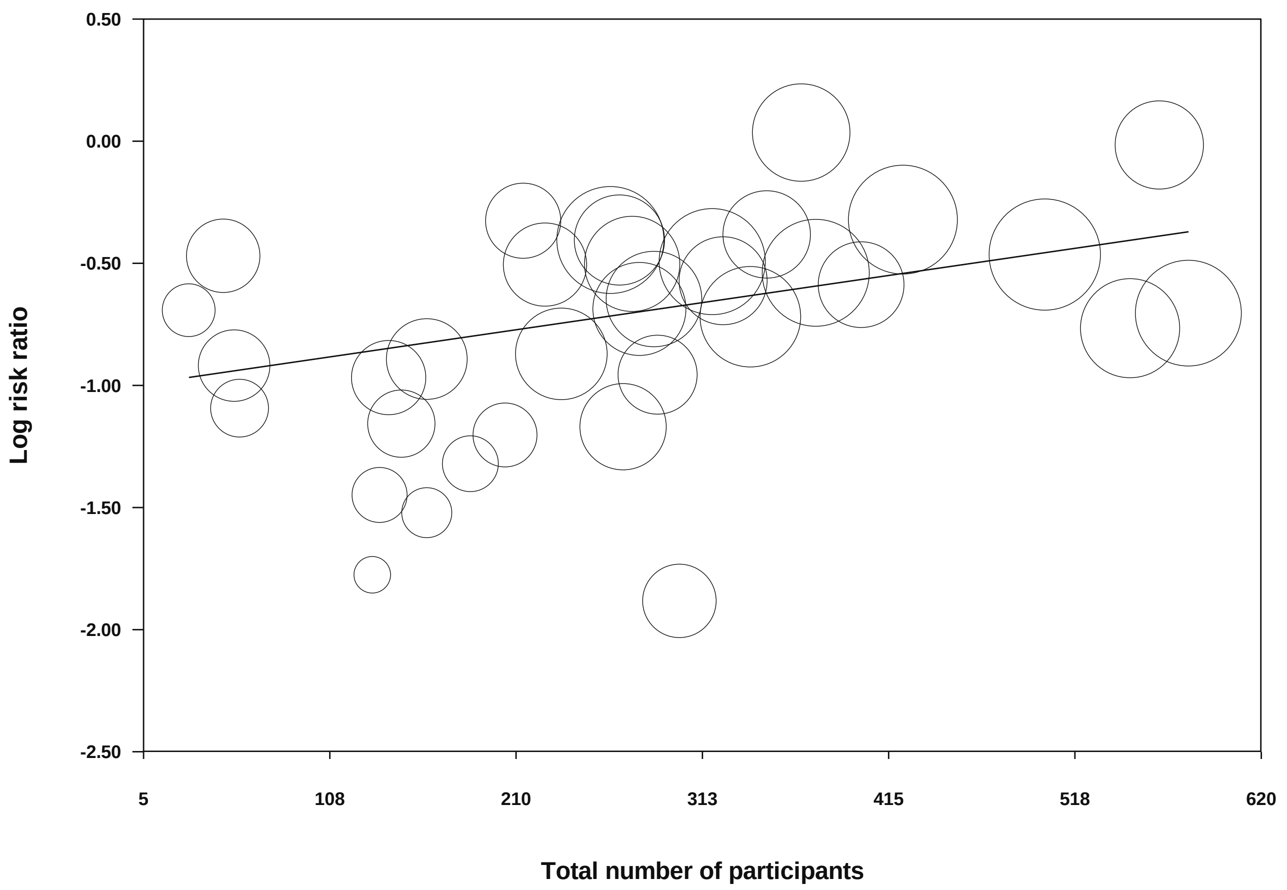

Figure S12-2. For event rate in each treatment group

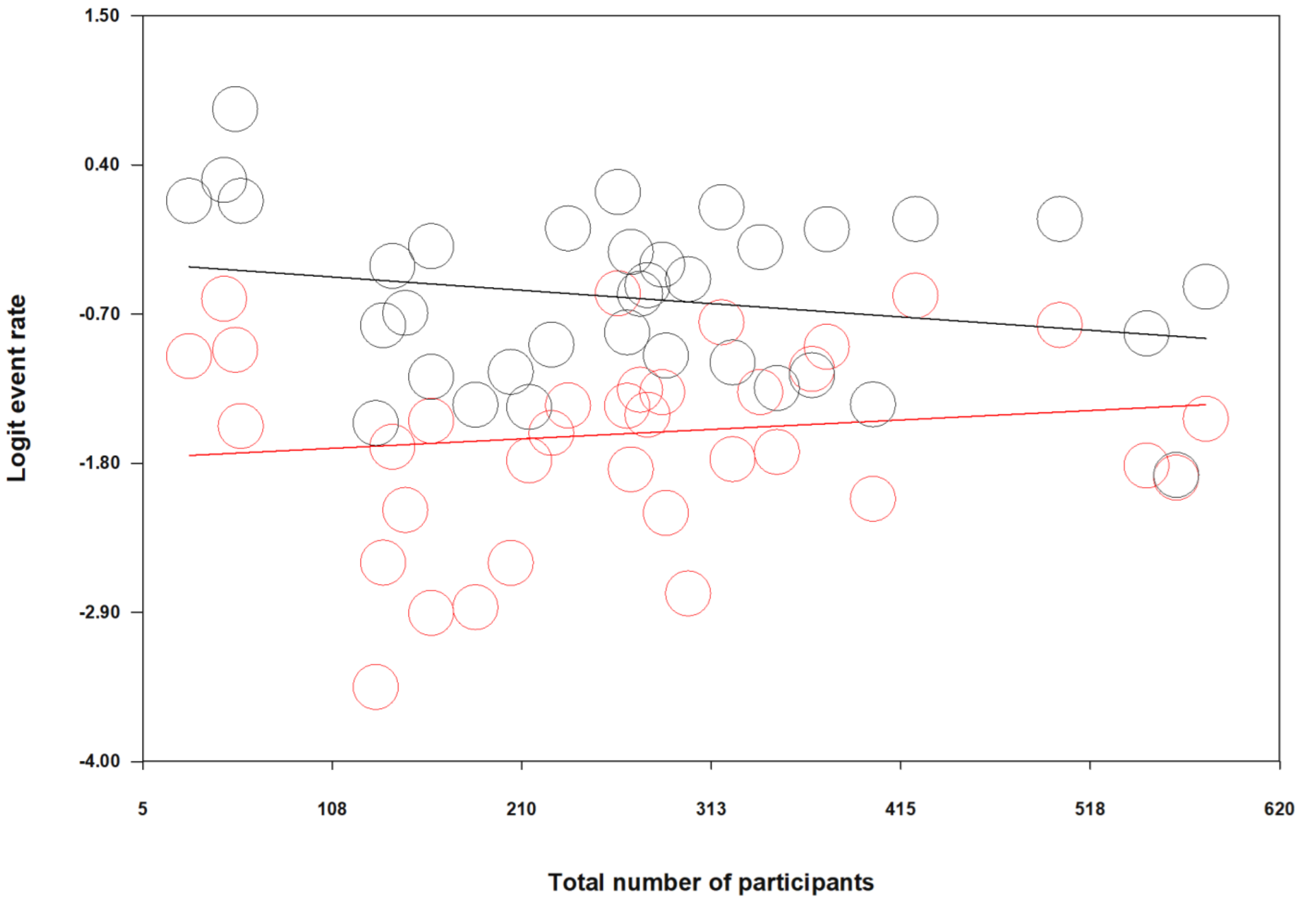

Red line, maintenance group; black line, discontinuation group

Figure S13. Bubble plot: dosing schedule

Figure S13-1. For effect size

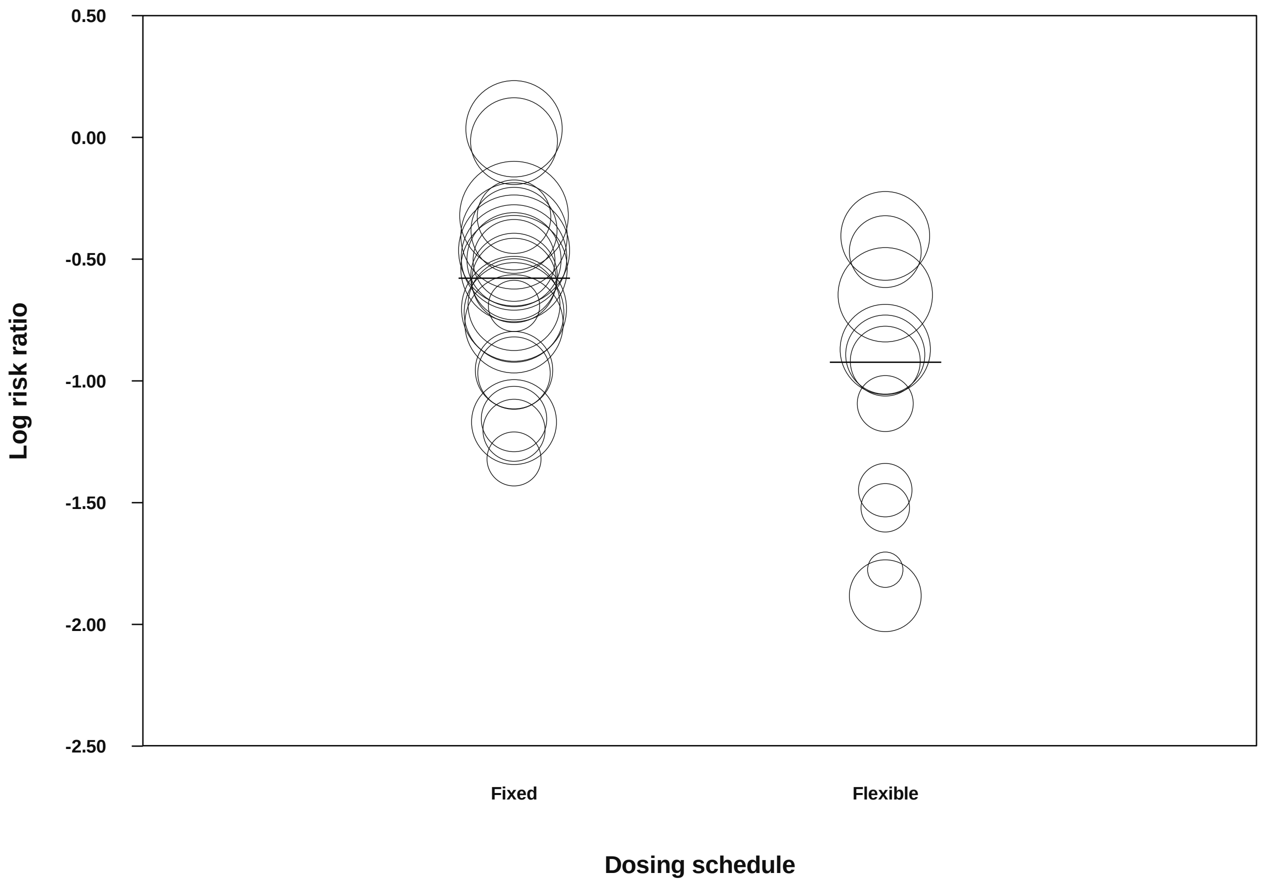

Figure S13-2. For event rate in each treatment group

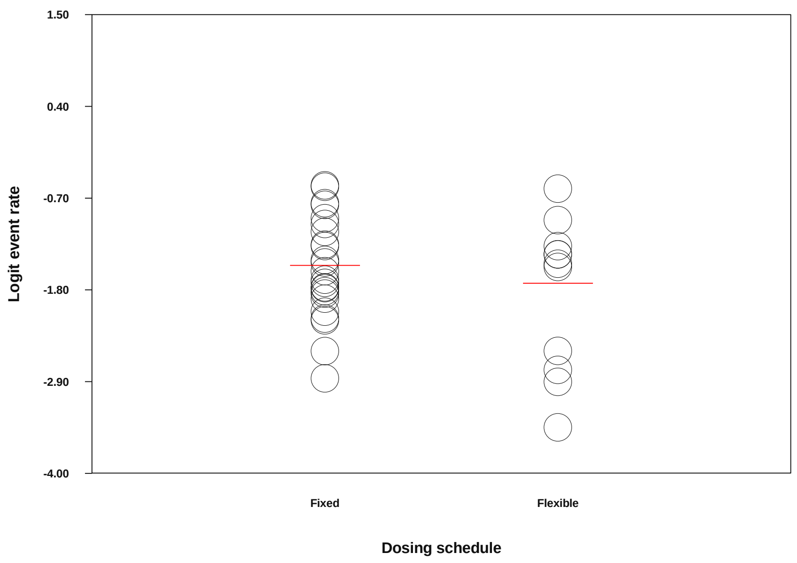

Maintenance group

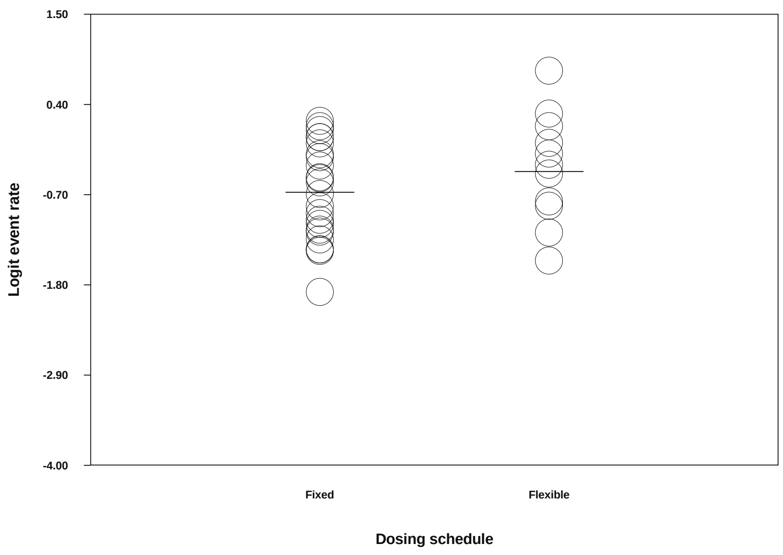

Discontinuation group

Figure S14. Bubble plot: drug class

Figure S14-1. For effect size

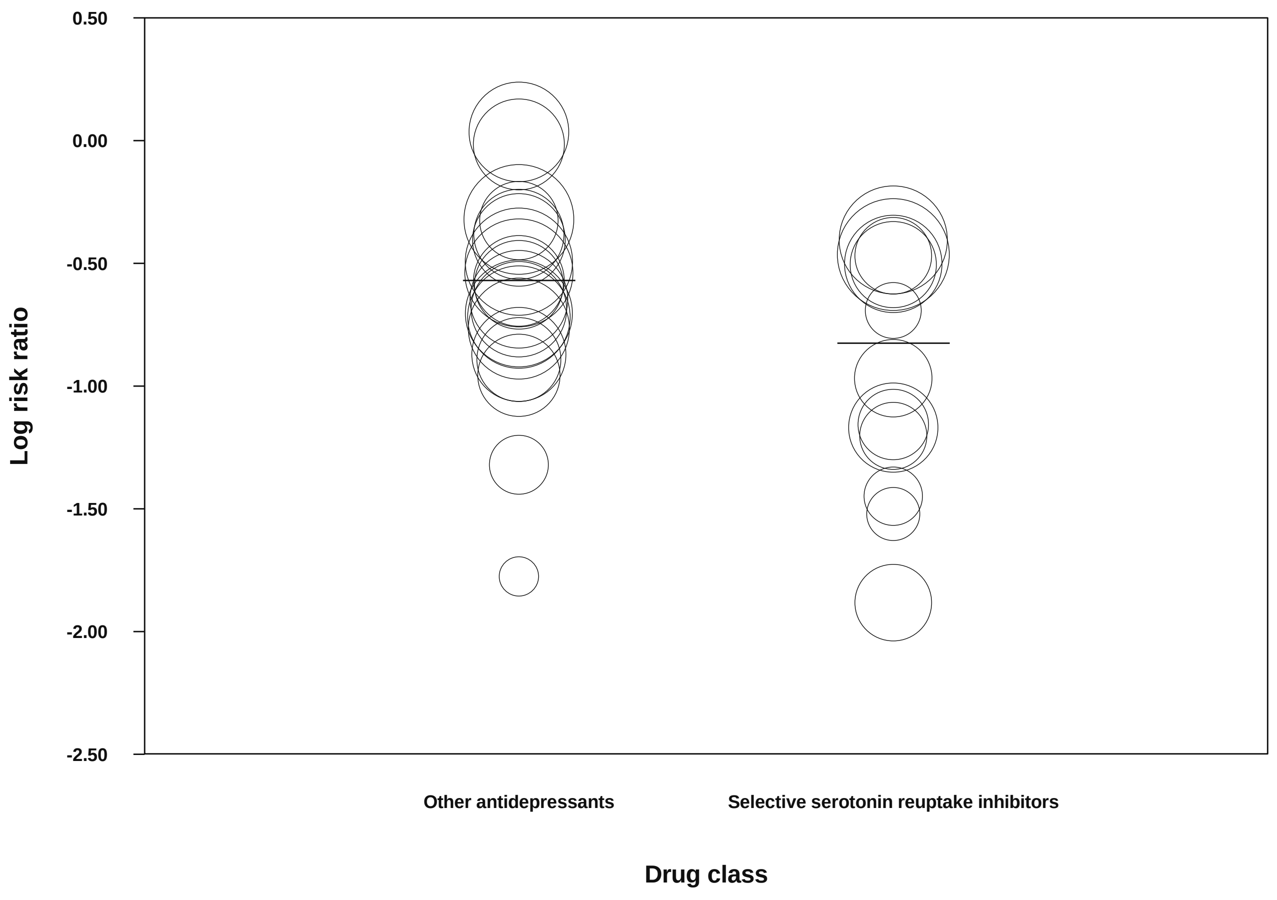

Figure S14-2. For event rate in each treatment group

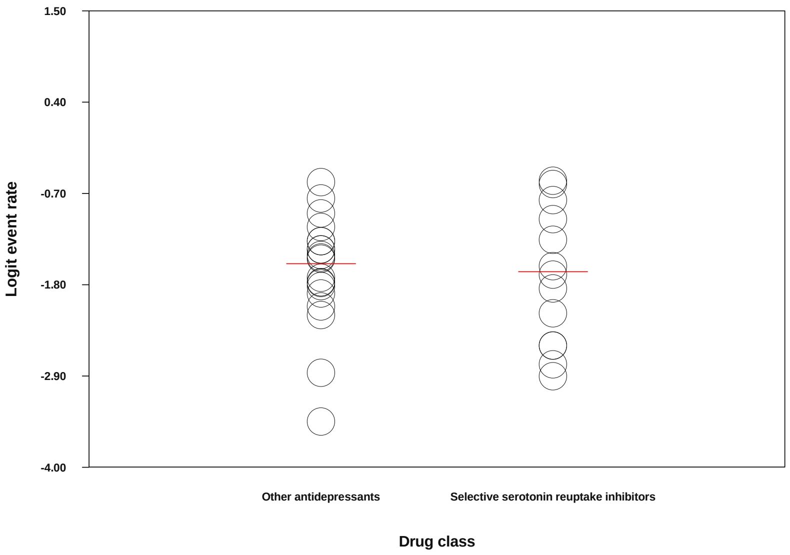

Maintenance group

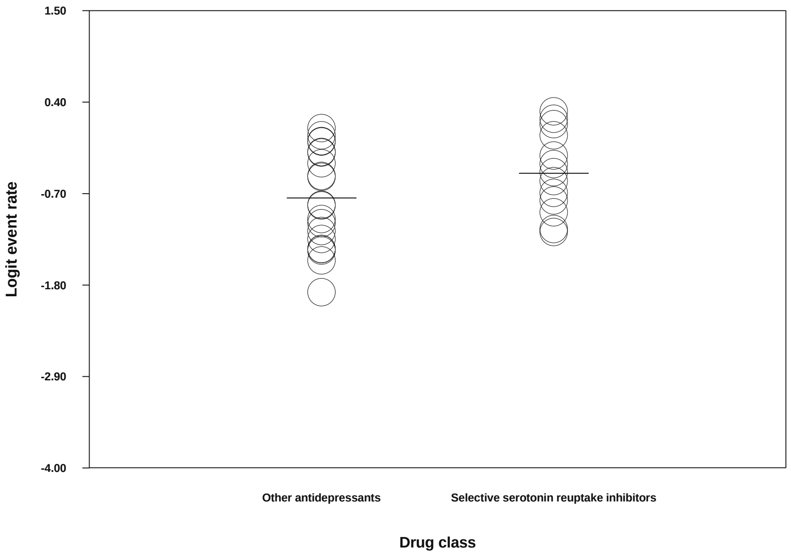

Discontinuation group

Figure S15. Bubble plot: publication year

Figure S15-1. For effect size

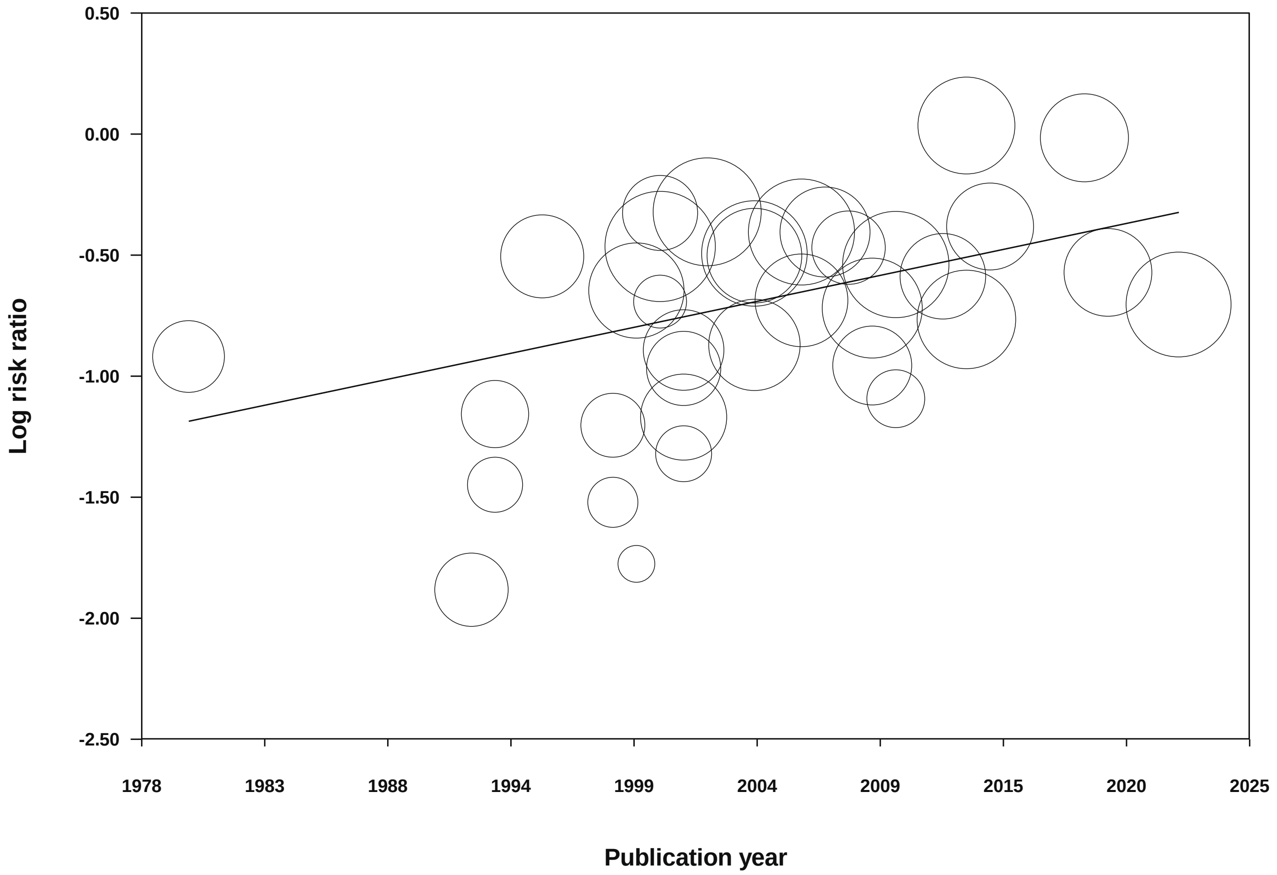

Figure S15-2. For event rate in each treatment group

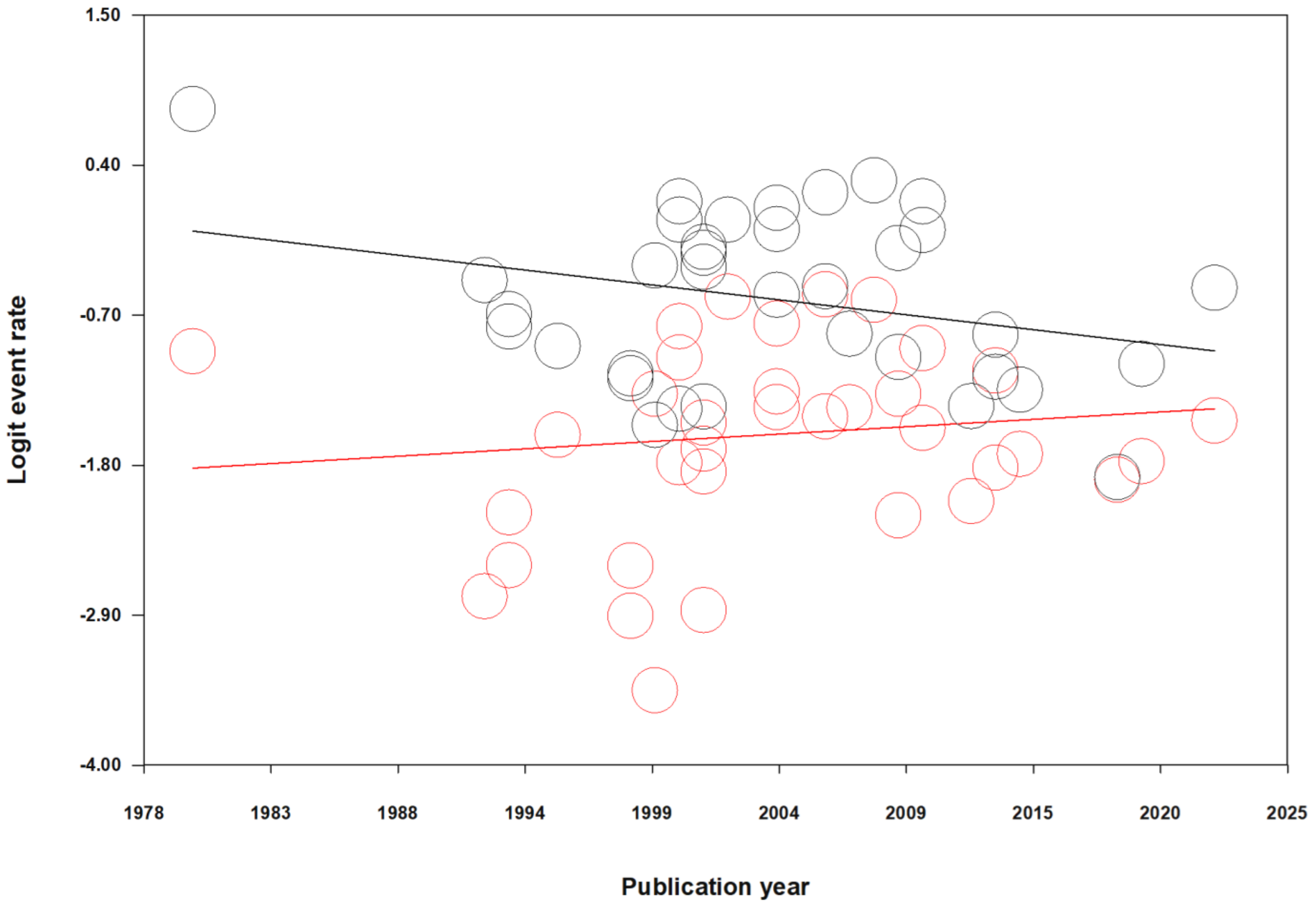

Red line, maintenance group; black line, discontinuation group

Figure S16. Evaluation of the publication bias

Figure S16-1. Funnel plot

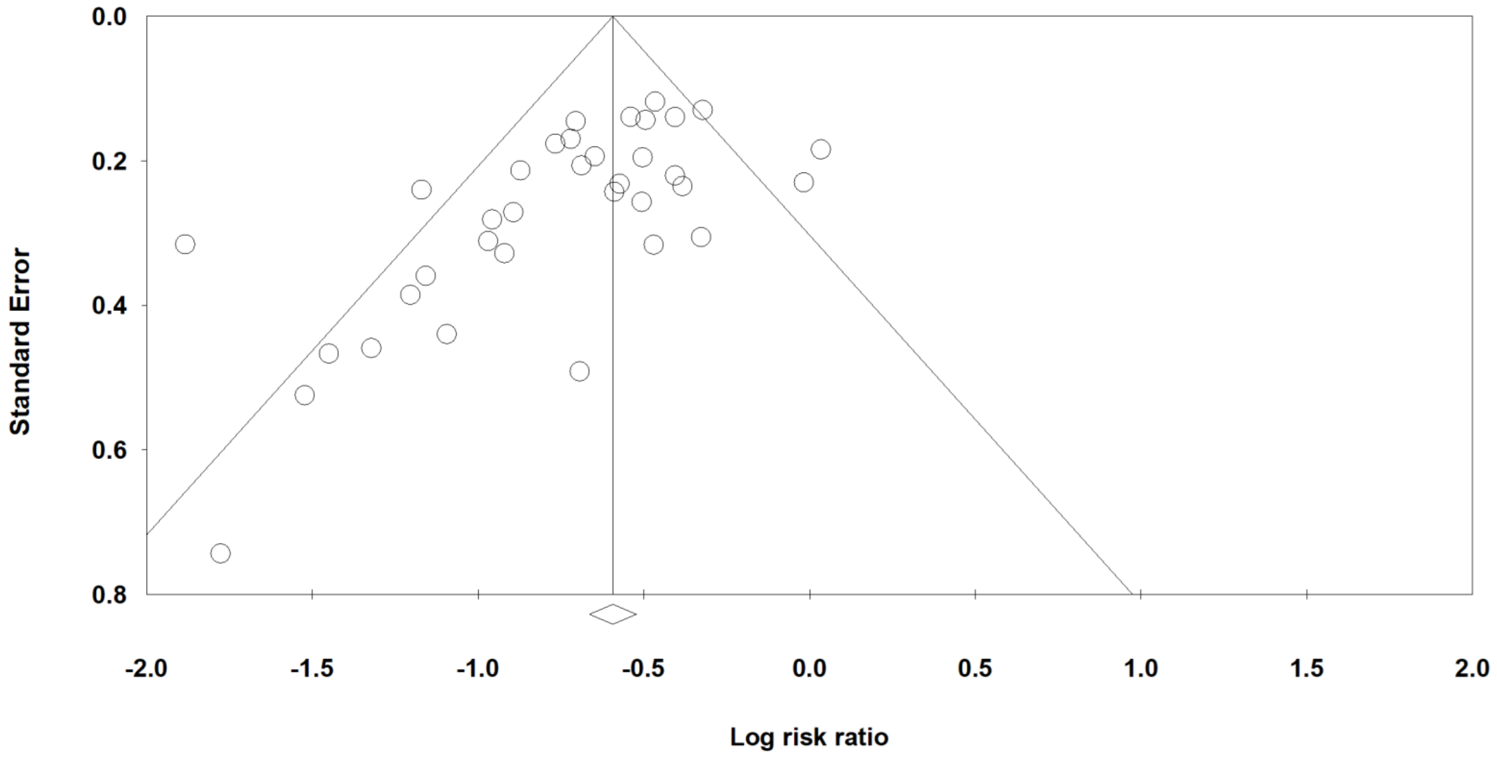

Figure S16-2. Egger's regression test

P = 0.0004

Table S1. PRISMA checklist

Section and Topic	Item #	Checklist item	Location where item is reported
**TITLE**			
Title	1	Identify the report as a systematic review.	P1
**ABSTRACT**			
Abstract	2	See the PRISMA 2020 for Abstracts checklist.	none
**INTRODUCTION**			
Rationale	3	Describe the rationale for the review in the context of existing knowledge.	P3
Objectives	4	Provide an explicit statement of the objective(s) or question(s) the review addresses.	P3
**METHODS**			
Eligibility criteria	5	Specify the inclusion and exclusion criteria for the review and how studies were grouped for the syntheses.	P4
Information sources	6	Specify all databases, registers, websites, organisations, reference lists and other sources searched or consulted to identify studies. Specify the date when each source was last searched or consulted.	P4
Search strategy	7	Present the full search strategies for all databases, registers and websites, including any filters and limits used.	P4
Selection process	8	Specify the methods used to decide whether a study met the inclusion criteria of the review, including how many reviewers screened each record and each report retrieved, whether they worked independently, and if applicable, details of automation tools used in the process.	P4
Data collection process	9	Specify the methods used to collect data from reports, including how many reviewers collected data from each report, whether they worked independently, any processes for obtaining or confirming data from study investigators, and if applicable, details of automation tools used in the process.	P4
Data items	10a	List and define all outcomes for which data were sought. Specify whether all results that were compatible with each outcome domain in each study were sought (e.g. for all measures, time points, analyses), and if not, the methods used to decide which results to collect.	P4
	10b	List and define all other variables for which data were sought (e.g. participant and intervention characteristics, funding sources). Describe any assumptions made about any missing or unclear information.	P4
Study risk of bias assessment	11	Specify the methods used to assess risk of bias in the included studies, including details of the tool(s) used, how many reviewers assessed each study and whether they worked independently, and if applicable, details of automation tools used in the process.	P4
Effect measures	12	Specify for each outcome the effect measure(s) (e.g. risk ratio, mean difference) used in the synthesis or presentation of results.	P4
Synthesis methods	13a	Describe the processes used to decide which studies were eligible for each synthesis (e.g. tabulating the study intervention characteristics and comparing against the planned groups for each synthesis (item #5)).	P4
	13b	Describe any methods required to prepare the data for presentation or synthesis, such as handling of missing summary statistics, or data conversions.	P4
	13c	Describe any methods used to tabulate or visually display results of individual studies and syntheses.	P4
	13d	Describe any methods used to synthesize results and provide a rationale for the choice(s). If meta-analysis was performed, describe the	P4

Section and Topic	Item #	Checklist item	Location where item is reported
		model(s), method(s) to identify the presence and extent of statistical heterogeneity, and software package(s) used.	
	13e	Describe any methods used to explore possible causes of heterogeneity among study results (e.g. subgroup analysis, meta-regression).	P4
	13f	Describe any sensitivity analyses conducted to assess robustness of the synthesized results.	P4
Reporting bias assessment	14	Describe any methods used to assess risk of bias due to missing results in a synthesis (arising from reporting biases).	P4
Certainty assessment	15	Describe any methods used to assess certainty (or confidence) in the body of evidence for an outcome.	P4
**RESULTS**			
Study selection	16a	Describe the results of the search and selection process, from the number of records identified in the search to the number of studies included in the review, ideally using a flow diagram.	P5
	16b	Cite studies that might appear to meet the inclusion criteria, but which were excluded, and explain why they were excluded.	P5
Study characteristics	17	Cite each included study and present its characteristics.	P5
Risk of bias in studies	18	Present assessments of risk of bias for each included study.	P5
Results of individual studies	19	For all outcomes, present, for each study: (a) summary statistics for each group (where appropriate) and (b) an effect estimate and its precision (e.g. confidence/credible interval), ideally using structured tables or plots.	P5
Results of syntheses	20a	For each synthesis, briefly summarise the characteristics and risk of bias among contributing studies.	P5
	20b	Present results of all statistical syntheses conducted. If meta-analysis was done, present for each the summary estimate and its precision (e.g. confidence/credible interval) and measures of statistical heterogeneity. If comparing groups, describe the direction of the effect.	P5
	20c	Present results of all investigations of possible causes of heterogeneity among study results.	P5
	20d	Present results of all sensitivity analyses conducted to assess the robustness of the synthesized results.	P5
Reporting biases	21	Present assessments of risk of bias due to missing results (arising from reporting biases) for each synthesis assessed.	P5
Certainty of evidence	22	Present assessments of certainty (or confidence) in the body of evidence for each outcome assessed.	P5
**DISCUSSION**			
Discussion	23a	Provide a general interpretation of the results in the context of other evidence.	P5
	23b	Discuss any limitations of the evidence included in the review.	P5
	23c	Discuss any limitations of the review processes used.	P5
	23d	Discuss implications of the results for practice, policy, and future research.	P5
**OTHER INFORMATION**			

Section and Topic	Item #	Checklist item	Location where item is reported
Registration and protocol	24a	Provide registration information for the review, including register name and registration number, or state that the review was not registered.	P4
	24b	Indicate where the review protocol can be accessed, or state that a protocol was not prepared.	P4
	24c	Describe and explain any amendments to information provided at registration or in the protocol.	P4
Support	25	Describe sources of financial or non-financial support for the review, and the role of the funders or sponsors in the review.	P6
Competing interests	26	Declare any competing interests of review authors.	P7
Availability of data, code and other materials	27	Report which of the following are publicly available and where they can be found: template data collection forms; data extracted from included studies; data used for all analyses; analytic code; any other materials used in the review.	P8

From: Page MJ, McKenzie JE, Bossuyt PM, Boutron I, Hoffmann TC, Mulrow CD, et al. The PRISMA 2020 statement: an updated guideline for reporting systematic reviews. BMJ 2021;372:n71. doi: 10.1136/bmj.n71

For more information, visit: http://www.prisma-statement.org/

This systematic review and pairwise meta-analysis was conducted according to the Preferred Reporting Items for Systematic Reviews and Meta-Analyses (PRISMA) statement (**Table S1**).[1] The literature search, data extraction, and entry into a spreadsheet for analysis were simultaneously and independently conducted by at least two authors (TK, KS, MH, MO, and YM). Furthermore, the authors double-checked all data for accuracy. Any discrepancies between the authors were resolved by discussion with a third author (NI). The study was registered with Open Science Framework (https://osf.io/9wnze/).

Inclusion criteria and literature search strategy

We performed a systematic literature review in accordance with the PICO strategy (“Patients,” adults with MDD in the maintenance phase; “Intervention,” monotherapy of antidepressants; “Control,” placebo; “Outcomes,” see the following section). The inclusion criteria were as follows: (1) DBRPCTs lasting at least 12 weeks and (2) DBRPCTs with an enrichment design in which patients are stabilized on the antidepressant of interest during the open-label study and then randomized to receive the same antidepressant or a placebo. The exclusion criteria were as follows: (1) studies focusing on specific generations (e.g., children/adolescents or older individuals) because the efficacy and safety of antidepressants in children and older individuals differ from those in the general adult population,[2] (2) studies including individuals with a dual diagnosis of MDD and other disorders because these studies might result in heterogeneity,[2] and (3) continuation studies that have randomly assigned

individuals with acute symptoms to treatment groups because in this study, among adults with MDD who benefited symptomatically from antidepressant treatment, we compared the differences in the relapse rates between those who continued on same antidepressant and those who discontinued the antidepressant. **Figure S1** shows the information regarding the literature search.

Data synthesis and outcome measures

The outcomes were relapse rates at 3, 6 (primary), 9, 12, 15, and 18 months, all-cause discontinuation, and discontinuation due to adverse event. **Table S2** presents the definitions of relapse for each study. **Table S3** shows the results of the data synthesis.

Data extraction

The authors independently extracted data from all included studies. All analyses were based on the intention-to-treat or modified intention-to-treat principles. When the data required for the meta-analysis were incomplete, we contacted the original study investigators to obtain the unpublished data. However, we did not obtain any additional data through personal communication. We also searched for missing data in published systematic review articles. For studies in which the Kaplan–Meier survival curves were reported, we measured the relapse rates from the curves using a ruler to match the observational time points of relapse rate in each DBRPCT.

Meta-analysis methods

This pairwise meta-analysis used a random-effects model.[3] We calculated risk ratios (RRs) with 95% confidence intervals (95% CIs). We assessed the heterogeneity of the included studies using the I^2 statistics, considering that I^2 of ≥50% indicates considerable heterogeneity.[4] We also conducted a single-group summary meta-analysis to calculate the exact relapse rates and 95% CIs in both the maintenance and discontinuation groups. When the pairwise meta-analysis showed significant differences in the relapse rates between the treatment groups, the number needed to treat (NNTB) was estimated.

We conducted the meta-regression analyses to examine whether the differences in the characteristics of the participants, treatment, and/or study design influenced the effect size for the primary outcome (**Table S4**). The following moderators were involved:

1. Factors related to the participants: (1) average age, (2) total number of participants, (3) number of episodes, (4) proportion of female participants, and (5) patient status (outpatients vs. both inpatients and outpatients).

2. Factors related to the treatments: (6) dosing schedule (fixed dosing vs. flexible dosing), (7) drug class based on the Anatomical Therapeutic Chemical classification system [selective serotonin reuptake inhibitors (SSRIs) vs. other antidepressants],[5] (8) antidepressant dose (the Defined Daily Dose),[5] and (9) discontinuation method

(abrupt discontinuation vs. gradual discontinuation).

3. Factors related to the study design: (10) publication year, (11) sponsorship (industry sponsorship studies vs. non-industry sponsorship studies), (12) country (USA studies vs. non-USA or international studies), (13) duration of the preliminary phase before randomization, and (14) risk of bias (studies evaluated as a low overall risk of bias vs. other studies).

We also performed another meta-regression analysis to investigate the association between the relapse rate at 6 months in the maintenance group or the discontinuation group and above potential explanatory factors. First, we conducted a univariable meta-regression analysis investigating separately the effect of each potential moderator. Then, we performed a multivariable meta-regression analysis using a stepwise procedure in which only covariates that are statistically significant with a p-value of <0.05 are included in the model.

We performed all statistical analyses using the Comprehensive Meta-Analysis Software Version 3 (Biostat Inc., Englewood, NJ, USA). We assessed the methodological quality of the included studies according to the Cochrane risk-of-bias tool for randomized trials (ROB2) (https://www.riskofbias.info/welcome/rob-2-0-tool). Finally, we used funnel plots and Egger’s regression tests to detect publication bias.

1. Page MJ, McKenzie JE, Bossuyt PM, Boutron I, Hoffmann TC, Mulrow CD, Shamseer L, Tetzlaff JM, Akl EA, Brennan SE, Chou R, Glanville J, Grimshaw JM, Hrobjartsson A, Lalu MM, Li T, Loder EW, Mayo-Wilson E, McDonald S, McGuinness LA, Stewart LA, Thomas J, Tricco AC, Welch VA, Whiting P, Moher D. The PRISMA 2020 statement: an updated guideline for reporting systematic reviews. BMJ. 2021;372:n71.
2. Herrman H, Patel V, Kieling C, Berk M, Buchweitz C, Cuijpers P, Furukawa TA, Kessler RC, Kohrt BA, Maj M, McGorry P, Reynolds CF, 3rd, Weissman MM, Chibanda D, Dowrick C, Howard LM, Hoven CW, Knapp M, Mayberg HS, Penninx B, Xiao S, Trivedi M, Uher R, Vijayakumar L, Wolpert M. Time for united action on depression: a Lancet-World Psychiatric Association Commission. Lancet. 2022;399(10328):957-1022.
3. DerSimonian R, Laird N. Meta-analysis in clinical trials. Control Clin Trials. 1986;7(3):177-188.
4. Higgins J, Thomas J, Chandler J, Cumpston M, Li T, Page M, Welch V. Cochrane Handbook for Systematic Reviews of Interventions version 6.2. www.training.cochrane.org/handbook. 2021.
5. WHO. Defined Daily Dose (DDD). https://www.who.int/medicines/regulation/medicines-safety/toolkit_ddd/en/.

Table S2. The definition of relapse/recurrence

Study name	The definition of relapse/recurrence
Stein 1980	No detailed information
Doogan 1992	CGI-S ≥ 4
Montgomery 1993 CIT	MADRS ≥ 22
Montgomery 1993 PAR	(1) CGI-S ≥ 4, (2) deterioration of CGI by ≥ 2 points, (3) met DSM-III-R criteria for MDD of 2 weeks, (4) needed antidepressant, or (5) Present of depressive symptomatology for > 7 days
Robert 1995	(1) MADRS ≥ 25 and (2) clinical judgment
Keller 1998	(1) met DSM-III-R criteria for MDD during ≥ 3 weeks, (2) CGI-S ≥ 4, (3) CGI-I ≥ 3, and (4) deterioration of HAMD24 by ≥ 4 points
Terra 1998	(1) met DSM-III-R criteria for MDD or (2) suicide attempt or completed suicide
Feiger 1999	(1) HAMD17 ≥ 18 for 2 consecutive visits or (2) lack of efficacy
Versiani 1999	(1) HAMD21 ≥ 18 or (2) deterioration of HAMD scores by ≥ 50%
Dekker 2000	HAMD17 ≥ 14
Rouillon 2000	(1) met DSM-III-R criteria for MDE and (2) HAMD21 ≥ 18 with the need to treat the recurrence
Schmidt 2000	(1) met SCID-P criteria for MDE and (2) deterioration of CGI-S by ≥ 2 points
Dalery 2001	(1) HAMD17 ≥ 15 and/or CGI ≥ 4 or (2) clinical judgement
Gilaberte 2001	(1) met DSM-III-R criteria for MDD, (2) HAMD17 ≥ 18, or (3) CGI ≥ 4
Hochstrasser 2001	MADRS ≥ 22
Thase 2001	Clinical judgment
Weihs 2002	The need for treatment intervention
Montgomery 2004	CGI-S ≥ 4
Rapaport 2004	(1) MADRS ≥ 22 or (2) withdrawal due to lack of efficacy
Simon 2004	(1) met DSM-IV criteria for MDD and (2) CGI-S ≥ 4 for 2 consecutive visits or final CGI-S ≥ 4
Perahia 2006	(1) deterioration of CGI-S by ≥ 2 points, (2) met MINI criteria for MDD for 2 consecutive visits
McGrath 2006	CGI-I ≥ 3 for 2 consecutive weeks
Kocsis 2007	(1) HAMD17 > 12 with a reduction of HAMD17 scores from acute phase by ≤ 50% for 2 consecutive visits, (2) withdrawal,

	and (3) met DSM-IV criteria for MDD
Dobson 2008	(1) HAMD17 ≥ 14 for 2 successive weeks, or (2) Psychiatric status rating ≥ 5 for 2 successive weeks
Goodwin 2009	(1) HAMD17 ≥ 16, (2) withdrawal due to lack of efficacy, or (3) suicide attempt or completed suicide
Perahia 2009	(1) CGI-S ≥ 4 and met DSM-IV criteria for MDD for ≥ 2 weeks, (2) met re-emergence criteria for 3 consecutive visits or 10 re-emergence visits, or (3) withdrawal due to lack of efficacy
Rickels 2010	(1) HAMD17 ≥ 16 or CGI-I ≥ 6, or (2) withdrawal due to lack of efficacy
Segal 2010	Met DSM-IV criteria for MDD
Boulenger 2012	(1) MADRS ≥ 22, or (2) clinical judgment
Goodwin 2013	(1) HAMD17 ≥ 16, (2) withdrawal due to lack of efficacy, or (3) suicide attempt or completed suicide
Rosenthal 2013	(1) HAMD17 ≥ 16, (2) withdrawal due to lack of efficacy, (3) hospitalization for depression, or (4) suicide attempt or completed suicide
Shiovitz 2014	(1) MADRS ≥ 22 for 2 consecutive visits, (2) deterioration of CGI-I by ≥ 2 points for 2 consecutive visits, (3) withdrawal due to lack of efficacy, or (4) MADRS (item 10) ≥ 4
Durgam 2018	(1) MADRS ≥ 18 for 2 consecutive visits, (2) discontinuation due to lack of efficacy (needed medication switch and deterioration of CGI-S by ≥ 2 points), (3) hospitalization for depression
Durgam 2019	(1) deterioration of CGI-S by ≥ 2 points, (2) risk of suicide, (3) hospitalization for depression, (4) needed medication switch, (5) MADRS ≥ 18 for 2 consecutive visits
Thase 2022	1) MADRS ≥ 22, (2) lack of efficacy, (3) unsatisfactory treatment response

CGI: Clinical Global Impressions, CGI-I: Clinical Global Impression–Global Improvement, CGI-S: Clinical Global Impressions–severity of illness, DSM(R or TR): Diagnostic and Statistical Manual of Mental Disorders(Revision or Text Revision), HAMD: Hamilton Rating Scale for Depression, MADRS: Montgomery Åsberg Depression Rating Scale

In the International Classification of Diseases 11th Revision, recurrent depressive disorder is defined by a history of at least two depressive episodes with an interval of several months without substantial mood disturbance. In the present study, the term “relapse” is used for convenience rather than “recurrence” similar to the previous study,[1] because few studies in this meta-analysis included cases in which worsening of symptoms during the study period was considered a recurrence.

1. Kato M, Hori H, Inoue T, Iga J, Iwata M, Inagaki T et al. Discontinuation of antidepressants after remission with antidepressant medication in major depressive disorder: a systematic review and meta-analysis. Mol Psychiatry 2021; 26(1): 118-133.

Table S3. Data synthesis of relapse rates

Study name	3 months	6 months	9 months	12 months	15 months	18 months
Stein 1980		○				
Doogan 1992	○	○	○			
Montgomery 1993 CIT	○	○ (data at 24 weeks)				
Montgomery 1993 PAR	○	○	○	○		
Robert 1995	○	○ (data at 24 weeks)				
Keller 1998	○	○	○	○	○	○
Terra 1998	○	○	○	○		
Feiger 1999	○	○	○ (data at 36 weeks)			
Versiani 1999	○	○	○			
Dekker 2000	○	○ (data at 22 weeks)				
Rouillon 2000	○	○	○	○		
Schmidt 2000		○ (data at 25 weeks)				
Dalery 2001	○	○	○	○	○	○
Gilaberte 2001	○	○	○	○		
Hochstrasser 2001	○	○	○	○	○	○
Thase 2001	○	○	○			
Weihs 2002	○	○	○			
Montgomery 2004	○	○	○	○		
Rapaport 2004	○	○	○ (data at 36 weeks)			
Simon 2004	○	○				
Perahia 2006	○	○				
McGrath 2006	○	○	○	○		
Kocsis 2007	○	○	○	○		
Dobson 2008	○	○	○	○		
Goodwin 2009	○	○ (data at 24 weeks)				
Perahia 2009	○	○	○	○		
Rickels 2010	○	○				
Segal 2010	○	○	○	○	○	○
Boulenger 2012	○	○	○			
Goodwin 2013	○	○	○			
Rosenthal 2013	○	○				
Shiovitz 2014	○	○ (data at 24 weeks)				
Durgam 2018	○	○				
Durgam 2019	○	○				
Thase 2022	○	○				

For studies in which the Kaplan–Meier survival curves were reported, we measured the relapse rates from the curves using a ruler to match the observational time points of relapse rate in each DBRPCT.

Table S4. Study characteristics

Study name	Region	AD	Sponsor	PT status	Diagnosis	Total n	Mean age ±SD	Female (%)	Number of episodes	Duration of preliminary phase (w)	Duration of RCT phase (w)	Mean score at baseline of acute study	AD dose	Mean final dose (mg/d)	Dosing schedule	Discontinuation method
Stein 1980	USA	AMI	Academia	OP	DSM3	55	42.3 ± 12.8	65	NI	8	26	HAMD: 25.1**	100-150	NI	Flexible	AB
Doogan 1992	International	SER	Industry	OP	DSM3	300	51	69	NI	8	44	HAMD17: ≥ 17	50-200	69.3-82.1	Flexible	AB
Montgomery 1993a	UK	PAR	Industry	NI	DSM3R	135	47.09 ± 8.76	78.52	NI	8	52	HAMD21: 26.9	20-30	NI	Flexible	NI
Montgomery 1993b	International	CIT	Industry	Both	DSM3R	147	NI	NI	NI	6	24	MADRS: ≥ 22	20 or 40	30.86	Fixed	NI
Robert 1995	France	CIT	Industry	NI	DSM3R	226	NI	71.68	NI	8	24	MADRS: ≥ 25	20, 40 or 60	NI	Fixed	NI
Keller 1998	USA	SER	Industry	OP	DSM3R	161	41.63 ± 9.38	65.84	1.85	28	76	HAMD24: 24.9	50-200	146.1	Flexible	TAP
Terra 1998	France	FLUV	Industry	NI	DSM3R	204	44.73 ± 11.00	73.53	3.5	24	52	MADRS: ≥ 24	100	100	Fixed	NI
Feiger 1999	USA	NEF	Industry	OP	DSM3R	131	41.31 ± 10.98	71.76	1.60	16	36	HAMD: 24.3	100-600	412	Flexible	NI
Versiani 1999	International	REB	Industry	Both	DSM3R	286	42.86 ± 11.89	73.43	NI	6	46	HAMD21: 29.6	4-8	NI	Flexible	NI
Dekker 2000	Netherland	FLUO	Industry	OP	DSM3R	30	37 ± 10	61.9	NI	16	22	HAMD17: ≥ 14	20	20	Fixed	NI
Rouillon 2000	France	MIL	Industry	Both	DSM3R	214	45.33 ± 10.1	67.28	2.98	26	52	HAMD21: 25.1	100	100	Fixed	NI
Schmidt 2000	USA	FLUO*	Industry	OP	DSM4	501	41.47 ± 11.34	68.26	NI	13	25	HAMD17: ≥ 18	20	20	Fixed	AB
Dalery 2001	France	TIA	Industry	Both	DSM3R	185	43.31 ±	65.41	2.56	6	79	HAMD17: 23.3	37.5	37.5	Fixed	NI
Gilaberte 2001	Spain	FLUO	Industry	OP	DSM3R	140	44.1	78.6	2.45	32	52	HAMD17: 24	20	20	Fixed	NI

Hochstrasser 2001	International	CIT	Industry	Both	DSM4	269	43.1 ± 10.64	71.2	3.5	22-25	48-78 w	MADRS: 30.5	20, 40 or 60	33.94	Fixed	NI
Thase 2001	USA	MIR	Industry	NI	DSM4	161	40.41 ± 11.61	50.64	NI	8-12	40	HAMD17: 22.7	30-45	38.6	Flexible	AB
Weihs 2002	USA	BUP	Industry	NI	DSM4	423	39.65 ± 0.25	65.01	3.00	8	44	HAMD21: ≥ 18	300	290	Fixed	NI
Montgomery 2004	International	VEN	Industry	OP	DSM3R	235	43.65 ± 11.08	68.89	3.21	26	52	HAMD21: 25.2	100~200	132-152***	Flexible	TAP
Rapaport 2004	USA	ESC	Industry	OP	DSM4	274	42.53 ± 11.69	60.95	NI	8	36	MADRS: ≥22	10 or 20	NI	Fixed	NI
Simon 2004	NA	VEN	Industry	NI	DSM4	318	42.05 ±	64.38	NI	8	26	HAMD21: 24.5	75, 150 or 225	177–191	Fixed	TAP
Perahia 2006	International	DUL	Industry	NI	DSM4	278	45.24 ± 12.25	72.66	NI	12	26	HAMD17: 23.7	60	60	Fixed	TAP
McGrath 2006	USA	FLUO	Academia	NI	DSM4	262	38.2 ± 10.9	55.3	NI	12	52	HAMD17: 17.7****	40 or 60	45.8	Fixed	NI
Kocsis 2007	USA	VEN	Industry	OP	DSM4	267	42.3 ±	68	NI	36	52	HAMD17: 22.4	75-300	220.8	Flexible	TAP
Dobson 2008	USA	PAR	Academia	OP	DSM4	49	38.93 ± 10.04	78.2	1.12	16	52	HAMD17: 20.9	10-50	NI	Flexible	TAP
Goodwin 2009	International	AGO	Industry	OP	DSM4TR	339	43.25 ± 10.58	74.31	3.6	8 or 10	24	HAMD17: 27.0	25 or 50	NI	Fixed	AB
Perahia 2009	International	DUL	Industry	OP	DSM4	288	47.54 ± 12.54	71.53	4.2	34	52	HAMD17: 23.1	60-120	84.3	Fixed	TAP
Rickels 2010	International	DESV	Industry	OP	DSM4	375	42.75 ± 12.04	67.47	NI	12	26	HAMD17: 24.2	200-400	NI	Fixed	TAP
Segal 2010	Canada	Various	Academia	OP	DSM4	58	43.78 ± 11.57	68.97	4.85	28	78	HAMD17: 19.2	NI	NI	Flexible	TAP
Boulenger 2012	International	VOR	Industry	Both	DSM4TR	400	44.95 ± 12.24	63.13	2.1	12	24-64	MADRS: 32.3	5 or 10	8.53	Fixed	AB
Goodwin 2013	International	AGO	Industry	OP	DSM4TR	367	45.64 ± 10.3	77.92	4.4	8	42	HAMD17: 26.3	25	25	Fixed	NI

Rosenthal 2013	International	DESV	Industry	OP	DSM4	548	45.95 ± 13	71.35	2.12	20	26	HAMD17: 24.2	50	50	Fixed	TAP
Shiovitz 2014	USA and Canada	LEVOM	Industry	OP	DSM4TR	348	43.28 ± 12.25	57.97	4.77	12	24	MADRS: 30.7	40, 80 or 120	79	Fixed	TAP
Durgam 2018	International	VIL	Industry	OP	DSM4TR	564	45.25 ± 12.21	63.06	4.60	20	28	MADRS: 31.7	20 or 40	30.05	Fixed	TAP
Durgam 2019	USA	LEVOM	Industry	OP	DSM5	324	45.39 ± 13.46	67.28	5.2	20	26	MADRS: 32.2	40-120	NI	Fixed	TAP
Thase 2022	USA	VOR	Industry	OP	DSM4TR	580	45.1 ± 13.23	72.42	NI	16	28	MADRS: 33.9	5, 10 or 20	11.72	Fixed	AB

AB: abrupt discontinuation, AD: antidepressant, AGO: agomelatine, AMI: amitriptyline, Both: both outpatient and inpatient, BUP: bupropion, CGI-S: Clinical Global Impressions - severity of illness, CIT: citalopram, d: day, DESV: desvenlafaxine, DSM(R or TR): Diagnostic and Statistical Manual of Mental Disorders(Revision or Text Revision), DUL: duloxetine, ESC: escitalopram, FLUO: fluoxetine, FLUV: fluvoxamine, HAMD: Hamilton Rating Scale for Depression, LEVOM: levomilnacipran, MADRS: Montgomery Åsberg Depression Rating Scale, MIL: milnacipran, MIR: mirtazapine, n: number of patients, NEF: nefazodone, NI: not information, OP: outpatient, PAR: paroxetine, PT: patient, RCT: randomized controlled trial, REB: reboxetine, SD: standard deviation, SER: sertraline, TAP: tapering discontinuation, TIA: tianeptine, UK: United Kingdom, USA: United States of America, VEN: venlafaxine, VIL: vilazodone, VOR: vortioxetine, w: week

*Daily or once weekly

**This study did not report the detailed information that participants in the acute study had a requirement of a scale-derived minimum of symptoms at baseline.

***The dose was the mean dose during the study.

****This study reported that participants in the acute study did not have a requirement of a scale-derived minimum of symptoms at baseline

Table S5. The results of univariable meta-regression analysis for the relapse rate at 6 months

	Drug–placebo difference				Drug response			Placebo response		
	Coefficient	95% CI	p	I^2 (%)	Coefficient	95% CI	p	Coefficient	95% CI	p
Moderators with a significant effect on drug-placebo differences										
Average age (K = 33)	-0.05	-0.09, -0.00	**0.0324**	52.90	-0.16	-0.21, -0.10	**<0.0001**	-0.10	-0.17, -0.04	**0.0020**
Total number of participants (K = 35)	0.00	0.00, 0.00	**0.0095**	52.71	0.00	-0.00, 0.00	0.3536	-0.00	-0.00, 0.00	0.1679
Dosing schedule (K = 35)	-0.35	-0.61, -0.08	**0.0094**	50.89	-0.21	-0.66, 0.23	0.3454	0.25	-0.16, 0.66	0.2277
Drug class (K = 33)	-0.26	-0.51, -0.00	**0.0486**	57.76	-0.10	-0.52, 0.33	0.6598	0.30	-0.09, 0.68	0.1305
Publication year (K = 35)	0.02	0.01, 0.03	**0.0039**	53.69	0.01	-0.01, 0.03	0.3953	-0.02	-0.04, 0.00	0.0610
Moderators with a significant effect on drug-response and/or placebo-response but without a significant impact on drug-placebo differences										
Sponsorship (K = 35)	-0.05	-0.44, 0.35	0.8081	57.70	-0.79	-1.43, -0.15	**0.0151**	-1.02	-1.62, -0.42	**0.0008**
Country (K = 34)	0.14	-0.11, 0.39	0.2605	56.62	0.50	0.13, 0.87	**0.0085**	0.32	-0.06, 0.71	0.1021
Risk of bias (K = 35)	-0.18	-0.49, 0.13	0.2500	57.65	0.24	-0.25, 0.73	0.3347	0.53	0.08, 0.98	**0.0222**
Moderators without a significant effect on neither drug-response, nor placebo-response, nor drug-placebo differences										
Number of episodes (K = 19)	0.12	-0.05, 0.29	0.1532	60.65	0.06	-0.19, 0.32	0.6218	-0.05	-0.29, 0.19	0.6737
Proportion of female (K = 34)	-0.01	-0.03, 0.01	0.3061	56.39	-0.02	-0.05, 0.02	0.3439	-0.01	-0.04, 0.02	0.6914
Patient status (K = 27)	0.16	-0.20, 0.51	0.3803	59.13	0.40	-0.08, 0.88	0.1004	0.29	-0.22, 0.81	0.2655
Antidepressant dose (K = 31)	0.02	-0.09, 0.14	0.6765	58.06	0.11	-0.11, 0.33	0.3134	0.10	-0.08, 0.28	0.2759
Discontinuation method (K = 20)	0.18	-0.10, 0.47	0.2010	50.98	-0.00	-0.51, 0.51	0.9891	-0.33	-0.86, 0.20	0.2219
Duration of the preliminary phase (K = 35)	-0.01	-0.02, 0.01	0.4015	55.98	-0.02	-0.04, 0.00	0.1251	-0.01	-0.03, 0.01	0.2135

95% CI: 95% confidence interval, N: number of studies

The I^2 value of this outcome in the primary analysis was 56.65%

Table S6. Multivariable meta-regression models for the relapse rate at 6 months (stepwise procedure)

Table S6-1. Drug–placebo difference

Covariate	Coefficient	95% CI	P-value
Intercept	-47.67	-74.24, -21.10	**0.0004**
Average age (K = 33)	-0.07	-0.11, -0.02	**0.0016**
Publication year (K = 35)	0.02	0.01, 0.04	**0.0003**

The I^2 value of this outcome in the primary analysis was 56.65%. I^2 of this multivariable regression model was 40.95%.

Table S6-2. Drug response

Covariate	Coefficient	95% CI	P-value
Intercept	5.67	3.35, 8.00	**<0.0001**
Total number of participants (K = 35)	0.00	0.00, 0.00	**0.0122**
Average age (K = 33)	-0.17	-0.23, -0.12	**<0.0001**

Table S6-3. Placebo response

Covariate	Coefficient	95% CI	P-value
Intercept	3.16	0.34, 5.99	**0.0280**
Average age (K = 33)	-0.07	-0.14, -0.00	**0.0448**
Sponsorship (K = 35)	-0.78	-1.40, -0.16	**0.0135**

95% CI: 95% confidence interval, K: number of studies

Supplementary text

Firstly, we found considerable heterogeneity in the primary outcome in the meta-analysis (I^2=56.65%, **Figure S4**), possibly because the included studies used different definitions for relapse (**Table S2**). In the multivariable meta-regression models including average age and publication year as a covariate, because the I^2 value decreased (I^2=40.95%, **Table S6**), these factors might also cause considerable heterogeneity. Moreover, we detected a publication bias in the result of the primary outcome (**Figure S16**). Our meta-regression analysis showed that studies with fewer subjects were associated with larger effect sizes regarding the primary outcome than studies with more subjects (**Figure S12**). Therefore, our results of the primary outcome might include a small-study effect. Secondly, we were unable to perform a meta-analysis on those outcomes since data on the relapse rates at time points longer than 18 months of observation was insufficient. Therefore, it remains unclear at this time whether a longer period of antidepressant treatment for those individuals is necessary. Thus, the long-term efficacy, acceptability, and tolerability of drugs need to be determined. Finally, Sim and colleagues reported that psychotherapy had some evidence of long-term benefit, especially in patients with three or more previous episodes of MDD.[1] However, our study did not cover these important clinical issues that might affect treatment decision-making in routine clinical practice (e.g., monotherapy or combination of antidepressants with nonpharmacological treatments).

1. Sim K, Lau WK, Sim J, Sum MY, Baldessarini RJ. Prevention of Relapse and Recurrence in Adults with Major Depressive Disorder: Systematic Review and Meta-Analyses of Controlled Trials. Int J Neuropsychopharmacol 2015; 19(2).
